# Cell membrane-coated nanomaterials for cancer therapy

**DOI:** 10.1016/j.mtbio.2023.100633

**Published:** 2023-04-12

**Authors:** Shiying Zeng, Qinglai Tang, Minna Xiao, Xinying Tong, Tao Yang, Danhui Yin, Lanjie Lei, Shisheng Li

**Affiliations:** a Department of Otorhinolaryngology Head and Neck Surgery, The Second Xiangya Hospital, Central South University, Changsha, 410011, China; b Department of Hemodialysis, The Second Xiangya Hospital, Central South University, Changsha, 410011, China; c State Key Laboratory of Bioelectronics, School of Biological Science and Medical Engineering, Southeast University, Nanjing, 210096, China

**Keywords:** Cell membrane, Nanomaterials, Cancer therapy, Nanotechnology

## Abstract

With the development of nanotechnology, nanoparticles have emerged as a delivery carrier for tumor drug therapy, which can improve the therapeutic effect by increasing the stability and solubility and prolonging the half-life of drugs. However, nanoparticles are foreign substances for humans, are easily cleared by the immune system, are less targeted to tumors, and may even be toxic to the body. As a natural biological material, cell membranes have unique biological properties, such as good biocompatibility, strong targeting ability, the ability to evade immune surveillance, and high drug-carrying capacity. In this article, we review cell membrane-coated nanoparticles (CMNPs) and their applications to tumor therapy. First, we briefly describe CMNP characteristics and applications. Second, we present the characteristics and advantages of different cell membranes as well as nanoparticles, provide a brief description of the process of CMNPs, discuss the current status of their application to tumor therapy, summarize their shortcomings for use in cancer therapy, and propose future research directions. This review summarizes the research progress on CMNPs in cancer therapy in recent years and assesses remaining problems, providing scholars with new ideas for future research on CMNPs in tumor therapy.

## Graphical abstract

**Figure:**
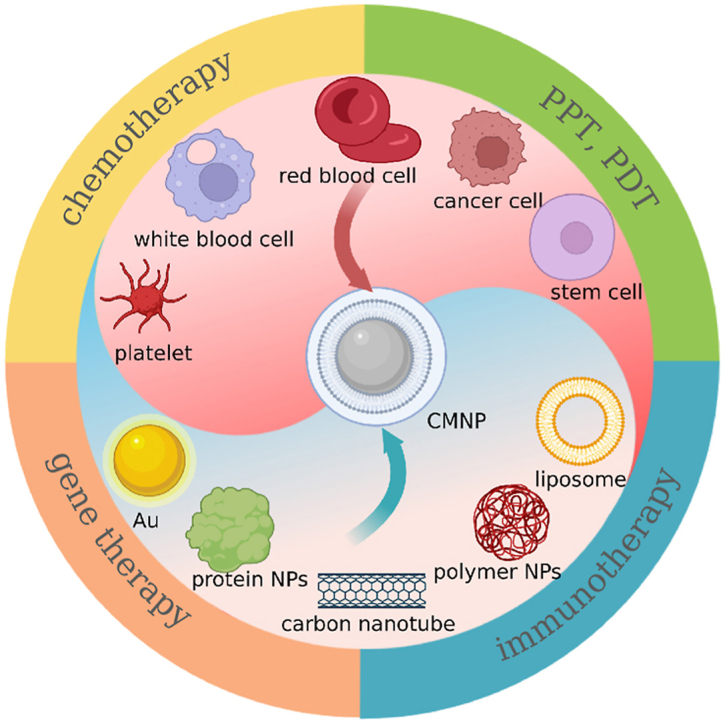

## 1 Introduction

Cancer is the second leading cause of human death and has been the focus of biomedical research and practice worldwide [[1], [2], [3]]. Cancer treatments mainly include surgery, radiotherapy, chemotherapy, and their combination, and targeted and biological therapies can be adopted when necessary. Surgery is more effective against early-stage cancers. For advanced cancer or tumors that occur in the vital organs, the tumors may not be completely removed, and the vital organs may be easily damaged, affecting the appearance and function and making the surgery less effective. Chemotherapy and radiotherapy are more toxic to tissues and organs, causing nausea, vomiting, fever, respiratory distress, bone marrow suppression, hair loss, and other symptoms, and have a long treatment period [4]. These complications will reduce treatment adherence, lead to treatment discontinuation, and affect patients’ quality of life [5]. New therapies, such as novel targeted therapy, oncolytic viral therapy, immunosuppressive therapy at immune monitoring sites, and chimeric antigen receptor T therapy has been used in clinical practice in recent years [[6], [7], [8], [9], [10], [11], [12]]. However, the newer technologies are not mature and may lead to drug resistance and other adverse reactions. The development of nanotechnology has largely solved the above problems, and various kinds of nanoparticles (NPs) have been introduced for cancer treatment.

NPs are new natural or artificially synthesized materials at nanometer diameters and comprising mainly inorganic and organic materials [13]. Through the enhanced permeability and retention effect (EPR) of solid tumors, NPs can passively and preferentially accumulate at tumor sites characterized by vascular leakage [14]. Moreover, NPs can well introduce proteins and other modifications, with high drug loading and protective effects on drugs [15,16]. NPs have been widely used in tumor therapy [[17], [18], [19]]. To prolong NP cycle time and better exploit their targeting specificity, surface modification technology is widely used to improve NP function. Nanoscale drug delivery systems (DDS) previously used for drug modification include liposomes, micelles, polymeric NPs, carbon nanotubes, and dendritic macromolecules [[20], [21], [22], [23]]. Although these DDS can improve the pharmacokinetics and biological distribution of drugs to some extent and have been validated in cellular and animal studies, they exert a certain toxicity due to their “exogeneity”. Some of them lack targeting, leading to the damage of normal cells, and are easily recognized by the immune system, shortening the half-life of drugs [[23], [24], [25]]. Simultaneously, DDS are not well absorbed by the tumor tissue and have a low targeting rate owing to the lack of physiological activity [24].

**Fig. 1:**
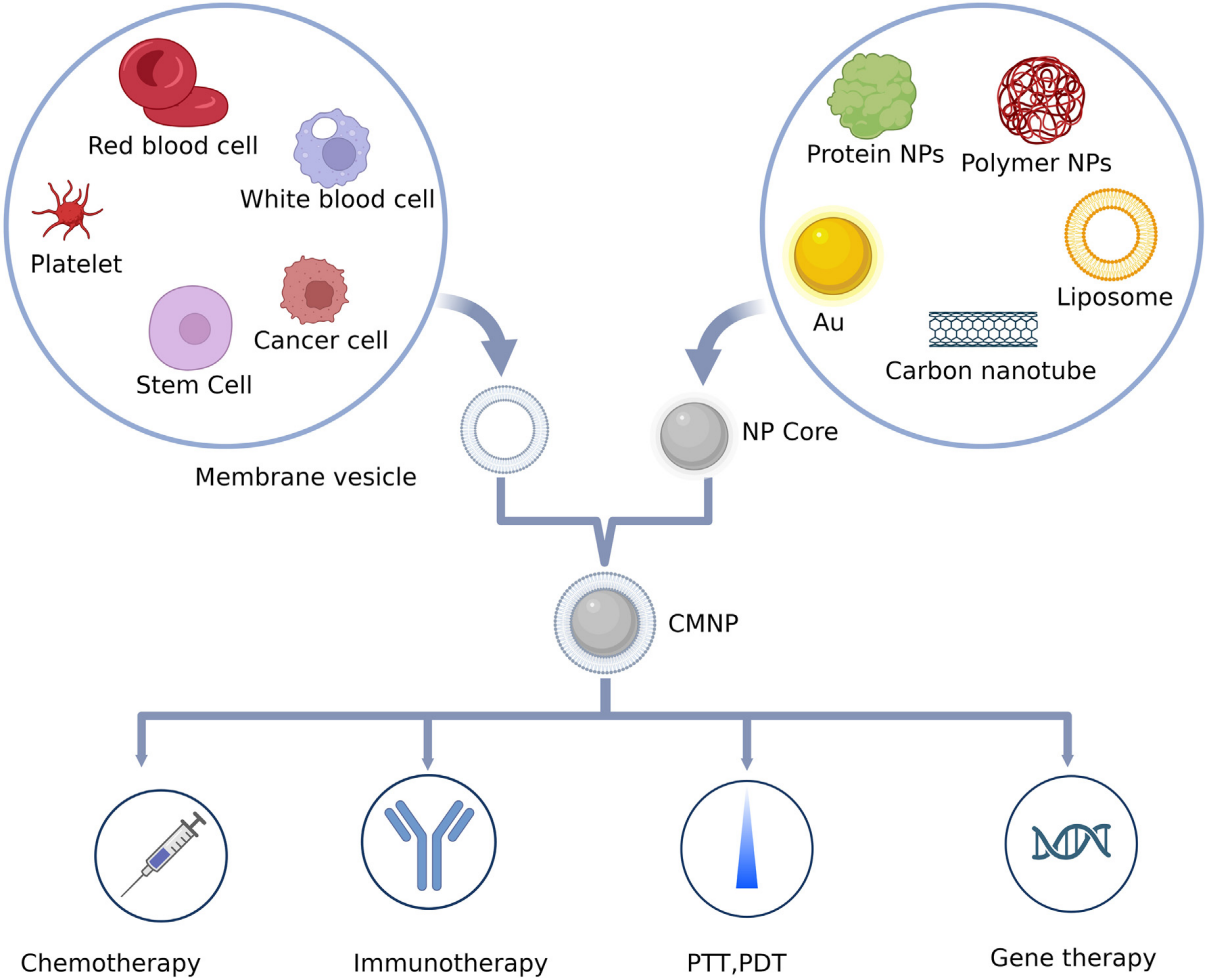
Cell-membrane coated nanoparticles for cancer therapy (created using BioRender.com.).

With the rapid development of nanotechnology and life sciences, surface modification methods for NPs are constantly updated. Especially the emergence of cell membrane-coated nanoparticles (CMNPs) technology in the recent years, which provides a new modification and camouflage strategy for the development of highly targeted and low immunogenic NPs, has been particularly important [[26], [27], [28], [29]], especially for tumor treatment and diagnosis [[30], [31], [32], [33], [34]]. CMNPs have some similar characteristics to cell membranes, such as immune evasion, long circulation time, and target recognition ability, which can avoid the disadvantages of other carriers; therefore, they can improve NPs biocompatibility, and confer adjustable surface properties [35]. Most previous studies focused on introducing the characteristics of CMNPs and researching their status. Here, we summarize the characteristics of different NPs and cell membranes, and display them in tables. Then, we summarize the synthesis process, preparation methods, and characterization of CMNPs and analyze the current research status and advantages of CMNPs for different types of cancer therapy, as well as the challenges that can be addressed by using CMNPs. Finally, we comprehensively assess the limitations of CMNPs. This review will provide medical workers and researchers with the latest research information, stimulating ideas to further our understanding of the application of CMNPs to cancer treatment. Fig. 1 summarizes the concepts covered herein.

## 2 Introduction to NPs

Their special physicochemical structure endows NPs with various properties, facilitating their use in new therapies for tumors. NPs have many advantages for tumor therapy, and rational design can maximize drug efficacy. For example, functionalized NPs can control drug release through different internal and external stimuli (such as pH, glutathione, light, and ultrasound), which may help prevent premature drug leakage in healthy tissues [36]. Organic NPs include common naturally occurring species, including lipoproteins, viruses, and ferritins, and synthetic NPs, including liposomes, protein-like dendrimers, emulsions, aptamers, solid lipid NPs, nanosomes, and other polymers [37,38]. These NPs are characterized by low cytotoxicity and high biocompatibility [39]. Inorganic NPs mainly include metal-like materials such as gold, silver, magnetic iron, nanohydroxyapatite, layered bimetallic hydroxides, and nonmetallic NPs such as mesoporous dioxide and carbon nanomaterials [13]. Inorganic NPs have good biocompatibility, high drug loading rate, ease of synthesis, and a wide range of possible surface conjugation chemistry [40]. These inorganic NPs have great potential for drug and gene delivery. The characteristics of common NPs used as cores for CMNPs in cancer treatment are shown in Table 1.

**Table 1:** Common materials used as cores of CMNPs for cancer therapy.

NP types	Advantages	limits	Refs
Liposome	High drug loading, biocompatibility, Easy to synthesize	Low stability	[74,75]
Protein NPs	Biocompatibility, functional groups, inherent biological activity, Immunogenicity	Low overall yield	[76]
PLGA	Large drug load, biocompatibility	Low targeting rate	[77,78]
Carbon-based NPs	Light weight, very high surface area, durability, and their diverse applications	Cytotoxic effects, low drug loading capacity	[58,59]
Magnetic NPs	Target therapy, controlling drug release, biosensing	Low biocompatibility, Potential organ toxicity	[79,80]
Mental NPs and Other iron oxide NPs	Enhancing agents, MRI contrast, Photothermal effect	Potential organ toxicity	[61,81]
Silica	Large drug load	Potential organ toxicity	[67,68]
Upconversion NPs	Luminescent properties, agnetic separation	Lack of biomedical applications, Potential organ toxicity	[70,71]

CMNPs: cell membrane coated nanoparticles, NPs: Nanoparticles. PLGA: poly (lactic glycolic acid).

### 2.1 Organic NPs

#### 2.1.1 Lipid NPs

Liposomes are a common class of lipid NPs and are circular soft-matter vesicles formed from one or more bilayer membranes, which include natural or synthetic phospholipids. They have amphiphilic affinity and can be easily surface-modified for drug delivery [41]. Liposomes are often used as carriers for the delivery of chemotherapeutic drugs, and several liposomal drugs are currently available for clinical use. The anticancer drug emtansine was loaded into pH-responsive liposomes and covered by macrophage (*M*ø) membranes, which improved targeting ability and inhibited lung metastasis [42]. Modifying NPs with specific liposomes increased their uptake by cancer cells. Zhang et al. [43] combined a modified liposomal membrane with a cancer cell membrane and used this hybrid membrane to encapsulate NPs to treat a mouse model of non-small cell lung cancer. The NPs to accumulated in tumors and inhibited tumor growth more effectively with long blood retention time and homologous targeting ability. Solid lipid NPs have low solubility in water and require surfactants for use. Solid lipid NPs contain triglycerides, lipids, fatty acids, steroids, and waxes. They can be released at specific times and delivered to the target site through food, injection, or other means [44].

#### 2.1.2 Protein NPs

Proteins are biological organic substances with complex four-dimensional structures composed of peptide chains. Owing to their biological origin, proteins have good biocompatibility and biological activity, low immunogenicity, and high drug-loading ability and stability. These characteristics are advantageous for use as NPs. Protein NPs commonly used in tumor therapy include albumin, ferritin, whey protein, lipoproteins, and silk protein [45]. By combining human serum albumin (HSA) with croconine (Croc), Chen et al. [46] prepared NPs by combining human serum albumin with croconine, forming a pH responsive photothermal agent, and demonstrated the effective photothermal ablation of large tumors. Wang et al. [47] synthesized ultra-small copper sulfide in ferritin nano-cage, and demonstrated its usefulness in photothermal treatment (PTT) effect.

#### 2.1.3 Polymer-assisted NPs

Polymer-assisted NPs are the most commonly used drug carrier NPs. When used as drug carriers, polymer-assisted NPs should be biocompatible and non-toxic, and with suitable physical structures. Furthermore, polymers should be chosen based on their half-lives without any leaking impurities and be mostly biodegradable. The advantages of polymer-assisted NPs include their high stability and mass production ability [13]. Polymer-assisted NPs used as cancer therapeutics include core-shell polymeric NPs, dendrimers, polymersomes, polyplexes, and nanogels [48]. For example, the biodegradable polymer poly (lactic glycolic acid) (PLGA), a common polymer for NPs, is polycondensed from lactic acid and glycolic acid in different proportions. PLGA has been approved by the US Food and Drug Administration, and is widely used in clinical medicine [[49], [50], [51], [52]]. Luk et al. [53] used a PLGA core to encapsulate doxorubicin (DOX) and further camouflaged it with a red blood cell membrane (RBCM) through ultrasound, and used it to treat lymphoma in mice.

### 2.2 Inorganic NPs

#### 2.2.1 Carbon-based NPs

Carbon-based NPs are promising emerging therapeutic tools and carriers with low toxicity, stable chemical properties, cheap raw materials, strong drug adsorption, good biocompatibility, unique optical properties, and large specific surface areas. Some studies listed carbon-based NPs separately from organic and inorganic NPs [54], mostly because they exhibit special properties, such as conductivity, thermal adsorption, optical properties, and electron affinity [55]. As drug carriers, carbon-based NPs accumulate in tumors through the EPR effect and are widely used in cancer therapy, but their potential toxicity risk should be considered [56,57]. Carbon-based NPs commonly used in cancer therapy include carbon nanodots, carbon nanotubes, graphene oxide, and mesoporous carbon nanospheres [58,59] and are usually applied to PTT/Photodynamic therapy (PDT). Li et al. [60] synthesized sulfur-doped carbon dots, which improved the efficacy of PDT in the treatment of oral squamous cell carcinoma.

#### 2.2.2 Metal and their related NPs

Owing to their surface plasmon resonance effect, metal NPs have excellent optical properties with strong absorption in the visible region to the near-infrared region (NIR), and have wide application prospects in biomedical detection, disease diagnosis, and treatment. They are magnetic and photosensitive and show potential for tumor imaging and PTT and PDT [32]. Among them, gold was the earliest discovered and is the most studied and most widely used [61]. Most *in vivo* applications use the strong shielding ability of gold NPs against X-rays to achieve contrast enhancement during computed tomography and local radiotherapy sensitization of tumors [62]. Freitas et al. [63] used phthalocyanine-Au NPs to treat melanoma, which produced a synergistic effect of PTT and PDT, killing more than 90% of the melanoma cells. The surface plasmon resonance absorption of other types of noble metal NPs, such as silver NPs and palladium NPs, can also be used for PTT of tumors. Magnetic NPs composed of iron, nickel, cobalt, and other metals and their oxides have a large specific surface area, which can easily be enriched and separated or targeted, and a magneto-thermal effect, which can indirectly kill tumor cells, and have broad application prospects in tumor therapy [64,65].

#### 2.2.3 Other inorganic NPs

Other inorganic NPs are also commonly used as carriers for nano drugs. Silica is considered an effective carriers because it has high biocompatibility and cane carry a large drug load because of its well-defined mesoporous and hollow structures [66]. However, silica may be toxic to the body [67,68]. Upconversion NPs (UPNPs) can convert NIR to visible light and have unique optical properties such as good photostability and narrow emission peaks and so can be used in fluorescence imaging. However, UPNPs lack application and may be toxic [[69], [70], [71]]. Prussian blue (PB) is also a common inorganic NPs, which is cheap, has low toxicity, show enzyme-like characteristics, and can be converted into Prussian white or Prussian green through oxidation–reduction reactions. Prussian blue is often used as a contrast agent in photoacoustic and magnetic resonance imaging (MRI) during clinical diagnosis and treatment and can be used as a photothermal agent in PTT. However, its biological behavior is still unclear, and its shape and specification for PTT need to be further explored [72,73].

### 2.3 Physicochemical properties of NPs

Owing to their smaller volume larger surface area, surface chemistry, composition and other properties, NPs can help drugs pass through various biological barriers in the body through EPR to reach tumor sites [82]. Other physicochemical properties of NPs also make them play various roles in tumor treatment, include catalytic, mechanical, magnetic, thermal, electronic and optical properties [54]. For example, Liu et al. [83] studied the thermal properties of gold NPs and their role in the PTT of tumors. However, these properties of NPs are not enough to exert the best therapeutic effect, and some NPs have drawbacks such as poor biological safety, short cycle time and poor tumor targeting. Surface modifications can improve their efficacy to some extent [24].

The size and shape of NPs also determines their toxicity [32,84], Some NPs may induce the body to produce excessive reactive oxygen species (ROS) [84], and excessive ROS can induce a series of pathophysiological effects. For example, compared with large particles, silver NPs have a larger surface area to volume ratio and so more easily come into contact with human lungs, blood and skin and enter the cell [85]. This results the damage to the mitochondrial through decreasing the ATP content of cells, and increasing the production of ROS in a dose-dependent manner [86]. Chen et al. [87] modified indocyanine green (ICG) with a cancer cell membrane (CCMICG) to carry out PTT and imaging of tumors. The results showed that the free ICG was relatively scattered and was cleared quickly, and the CCMICG showed better targeting and cycle time. CMNPs are mainly composed of functional NPs and bioactive cell membrane coatings, which plays a role in encapsulation, protection, and targeting to deliver functional NPs particles to the lesion sites [88].

## 3 Characteristics of different cell membranes

There are still many challenges and limitations in the application of NPs in tumor therapy. To overcome these challenges, cell membranes from various sources have been used. These cell membranes are important mediators of information exchange and signaling in living organisms, and the abundance of recognition units (e.g., proteins and glycans) on their surfaces confers a high degree of biological specificity. Good properties, such as long blood circulation, targeting ability, and immune escape, can be conferred to CMNPs from cell membranes [89]. There are many types of cell membranes used to encapsulate NPs, and each cell membrane has different advantages. This chapter focuses on several cell membranes that encapsulate NPs for cancer therapy. Table 2 shows the characteristics of cell membranes and some examples of their application in tumor therapy as CMNPs.

**Table 2:** Characteristics of cell membranes and some examples of their application in tumor therapy as CMNPs.

Membrane types	Advantages	Limits	Therapy type	Nanoparticle cores	Refs
Cancer cell	Homologous targeting, Immune escape, Antitumor response	Unknown pathogenicity	Chemotherapy, Immunotherapy, PDT, PTT, Gene therapy	PLGA-DOX, PLGA-R837, BQODs, SSAP-Ce6, PLGA-ICG, PLGA- siRNA & DOX	[87,94,[165], [166], [167], [168]]
Red blood cell	Long circulation (∼120 days), Good biocompatibility, Ability to transverse endothelium	Poor tumor-targeting, Simple preparation	Chemotherapy, Immunotherapy, PDT, PTT, Gene therapy	PLGA-DOX, BQODs, Fe_3_O_4_-ICG, Au NCs, Self-assemble NPs(siPgp/cADs)	[53,[169], [170], [171], [172], [173]]
Platelet	CTC-targeting, Low immunogenicity, Subendothelium binding, Inhibit the immune complement system attack	Small, Low blood concentration	Chemotherapy, Immunotherapy, PDT, PTT, Gene therapy	Nanovehicle-DOX and TRAIL, Fe3 O4 -SAS, PLGA NPs-verteporfin, Au nanorods MOF-siRNA	[24,108,113,[174], [175], [176], [177]]
Stem cell	Tumor-targeting, Tumor-homing affinity	High cost	Chemotherapy, Immunotherapy, PDT, PTT, Gene therapy	Polydopamine-siRNA-DOX. PLGA b-NaYF4:Yb3+, Er3+ UCNPs, Lipids-Au Nr-iron oxide-DOX, Polydopamine-siRNA-DOX	[119,[178], [179], [180], [181], [182], [183]]
White blood cell:	Tumor-targeting, Reducing opsonization, Transendothelial migration	High heterogeneity, Macrophages are nonproductive cells	Chemotherapy, Immunotherapy, PTT	PLGA-DOX, PLGA-IL2, PLGA-CFZ, Fe3O4 NPs	[137,141,[184], [185], [186]]
Hybrid cell membrane	Assemble multiple advantages	Function destruction	Chemotherapy, Immunotherapy, PDT, PTT, Gene therapy	Liposomal NPs-PTX, Mesoporous silica NPs-R873, liposome-Ce6, Melanin NPs, PLGA-siRNA	[152,[187], [188], [189], [190]]

PTT: photothermal therapy, PDT: photodynamic therapy; DOX: doxorubicin; PLGA: poly (lactic glycolic acid); BQODs: Black phosphorus quantum dots; SSAP: Polymer formed by styrene, acrylic acid and polyethylene imine; ICG: indocyanine green; Au NCs: Gold nanocages; SAS: sulfasalazine; UCNPs: Upconversion nanoparticles; R837: toll-like receptor 7 agonist, imiquimod; MOF: metal-organic framework; PDA: PD-L1 siRNA; CTC: circulating tumor cells.

### 3.1 Cancer cells

Cancer cells evolve from normal cells, which are stimulated by pathological states. Unlike normal cells, cancer cells are characterized by the presence of plasma membrane proteins on their surface, which allows them to proliferate indefinitely, adhere to isotypes, and effectively avoid clearance by the immune system [26,90]. Moreover, because cancer cells can replicate indefinitely and are easy to culture, their availability is greatly increased. Coating NPs with cancer cells membranes (CCMNPs) impart the characteristics of the membranes onto the drugs, including homologous adhesion and inherent immune escape, and enhance the recognition ability of drugs to target organs or target cells, which can improve the efficiency of drug delivery to tumors to a certain extent [31]. The unique characteristics exhibited by cancer cells can be largely explained by the presence of complex antigenic features on their membranes, which enable cancer cells to recognize each other specifically, enabling homologous tumor cells to be close to each other [26,90]. Without the need for complicated chemical modifications, the cancer cell membrane can achieve the function of “localization” through its own adhesion and recognition of cancer cells, so that the CCMNPs can be enriched on the surface of cancer cells in the body, which greatly reduces the damage to the body's normal cells.

**Fig. 2:**
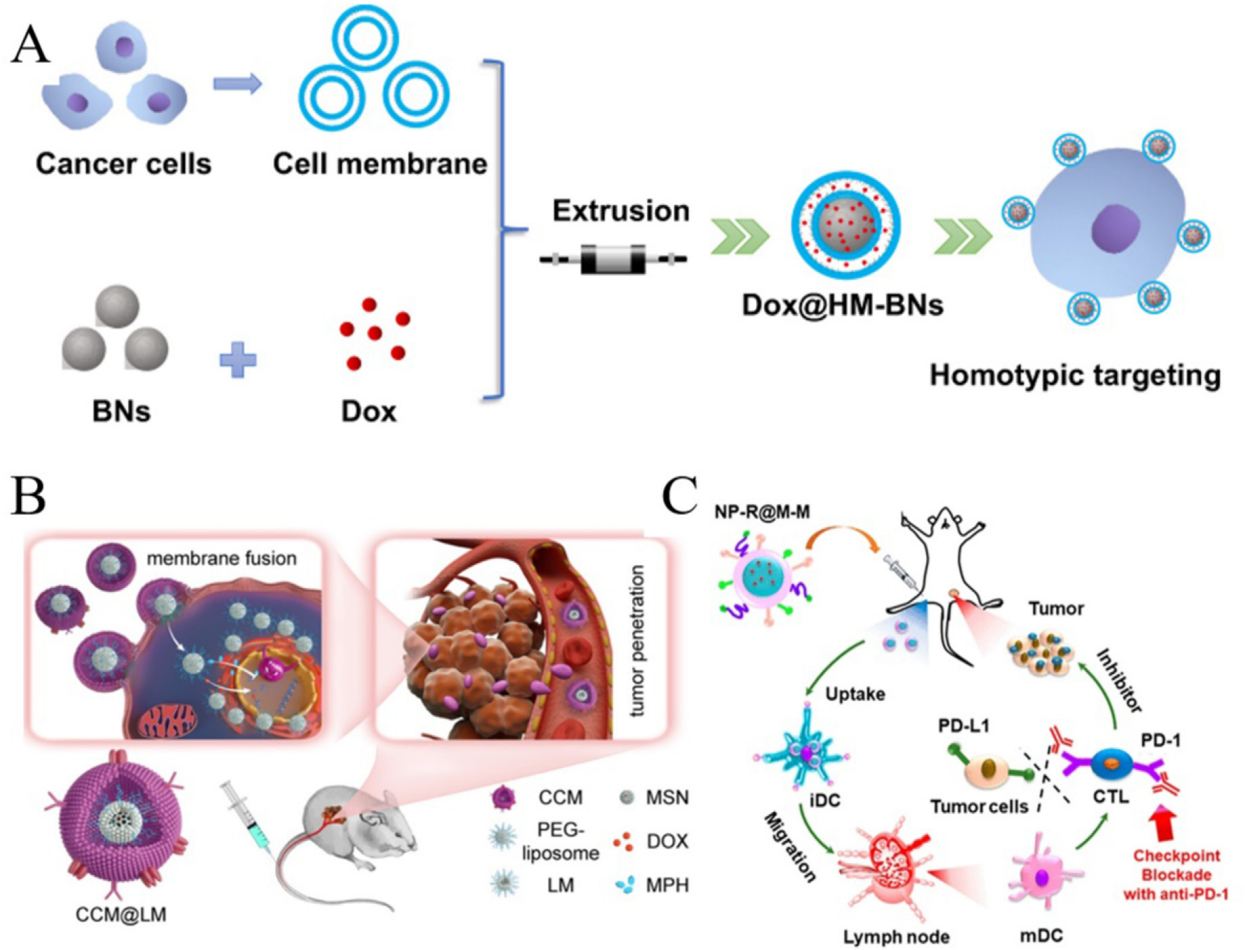
**Characteristics of cancer cell membranes and application of cancer cell membrane nanoparticles (CCMNPs) in cancer therapy. (A)** Synthesis process and homotypic targeting of CCMNPs; reproduced with permission from Creative Commons Attributes (CC BY NC) [91]. **(B)** CCMNPs in cancer chemotherapy; adapted and reproduced with permission from American Chemical Society [92]. **(C)** CCMNPs in anticancer vaccination; adapted and reproduced with permission from American Chemical Society [94].

CCMNPs have been extensively studied in cancer treatment. For example, Feng et al. [91]used the homologous targeting of cancer cell membranes to prepare CCMNPs for tumor chemotherapy, which is more effective than simple chemotherapy drugs. Cancer cell membranes encapsulated with low doses of DOX and a poly ADP-ribose polymerase inhibitor (mefurpiride hydrochloride) were used to treat breast cancer and showed potent antitumor effects (Fig. 2B) [92]. Zhao et al. [93] used cancer cell membrane-camouflaged mesoporous silica NPs, which were loaded with dacarbazine with anti-programmed cell death protein 1 antibody, combined with chemotherapy and immunotherapy provide a promising nanoplatform with potential applications for the treatment of melanoma.Using the antigen presentation characteristics of cancer cell membranes, Yang et al. [94] developed the CCMNPs as an anticancer vaccination and to provide immunotherapy for melanoma (Fig. 2C). In addition, owing to their unique properties, CCMNPs are also commonly used to study of targeted chemotherapy [95,96], PTT, PDT, and acoustodynamic therapies [[97], [98], [99]].

### 3.2 Red blood cells

**Fig. 3:**
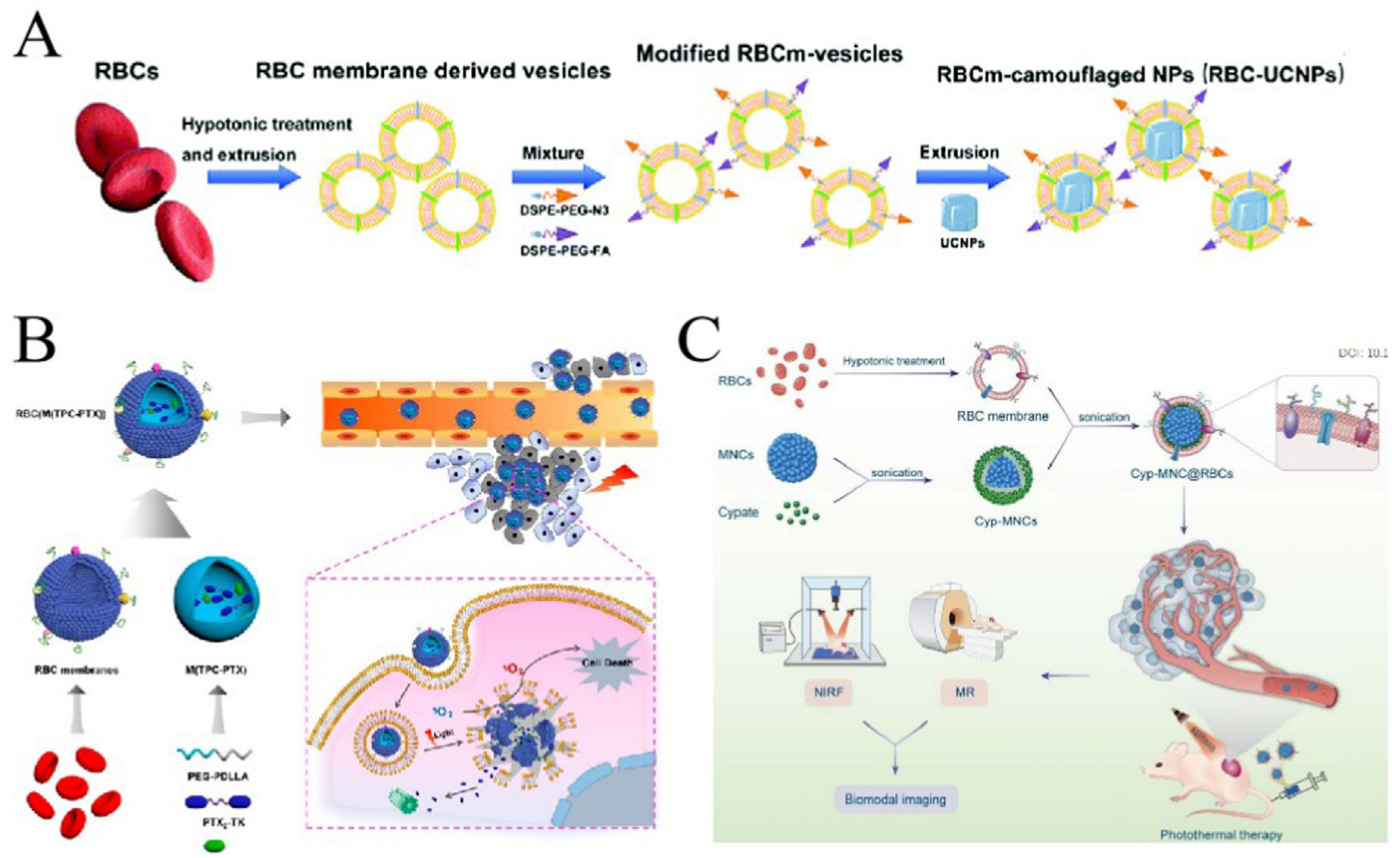
Characteristics of red blood cell membranes (RBCMs) and application of RBCM-coated nanoparticles (RBCMNPs) in cancer therapy. (A) Modified RBCM has good targeting ability; adapted and reproduced with permission from Royal Society of Chemistry [103]. **(B)** RBCMNPs in combination with photodynamic- and chemotherapy; adapted and reproduced with permission from American Chemical Society [104]. **(C)** RBCMNPs in photothermal therapy; adapted and reproduced with permission from Royal Society of Chemistry [105].

RBCs are the most abundant blood cells in the blood and contain hemoglobin, and primary medium for transporting oxygen through the blood, and have an immune function. Because RBCs circulate in the blood for approximately 120 days, they are very good carrier cell membranes for long-circulating drugs. They are also the first cell membranes to be used in nanodrug delivery systems [100]. Owing to surface immune marker molecules such as CD47, RBCs can avoid phagocytosis by Mø in circulation, which lends RBC membranes (RBCMs) good biocompatibility and non-immunogenicity [101]. RBCs do not contain nucleus, so extracting the membrane is relatively easy, and the membrane properties can be largely preserved. However, owing to their lack of surface adhesion molecules, RBCs lack the corresponding targeting ability. This drawback can be compensated for direct modification (combining a ligand with the RBCMs containing an active group) or indirect modification (inserting a positively charged ligand on the membrane) [102]. Li et al. [103] enhanced the tumor targeting ability of RBCM-coated NPs (RBCMNPs) by modifying RBCM, i.e., combining 1,2-distearoyl-*sn*-glycero-3-phosphoethanolamine-N- [folate (polyethylene glycol)-2000] (DSPE-PEG-FA] with cell membrane (Fig. 3A).

RBCMNPs have also been extensively studied. Pei et al. [104] used RBCMs to encapsulate photosensitizers and ROS-reactive PTX dimers. The obtained RBCMNPs were used for synergistic chemotherapy and PDT of tumors (Fig. 3B). The coating of the RBCM prolongs blood circulation and improves the involvement of drugs in tumors. Wang et al. [105] wrapped superparametric nanoclusters (MNCs) with RBCMs (Fig. 3C). The obtained MNC@RBCs were used for tumor imaging and PTT. They were more stable than bare MNCs and also exhibited stronger tumor-homing abilities. Furthermore, Daniyal et al. [106] prepared a RBCMNP for combination with chemotherapy-phototherapy for cervical cancer. They used RBCMs encapsulating Prussian blue NPs loaded with J5 natural compound extracted from lanceolate crescent fern and modified the surface of the compound with folic acid. The modified RBCMNPs showed stronger tumor accumulation than RBCMs. The above research shows that RBCMNPs effectively prolong the circulation time of NPs *in vivo* and reduce their toxicity and side effects, and modified RBCMs can also achieve good targeting.

### 3.3 Platelet membrane

**Fig. 4:**
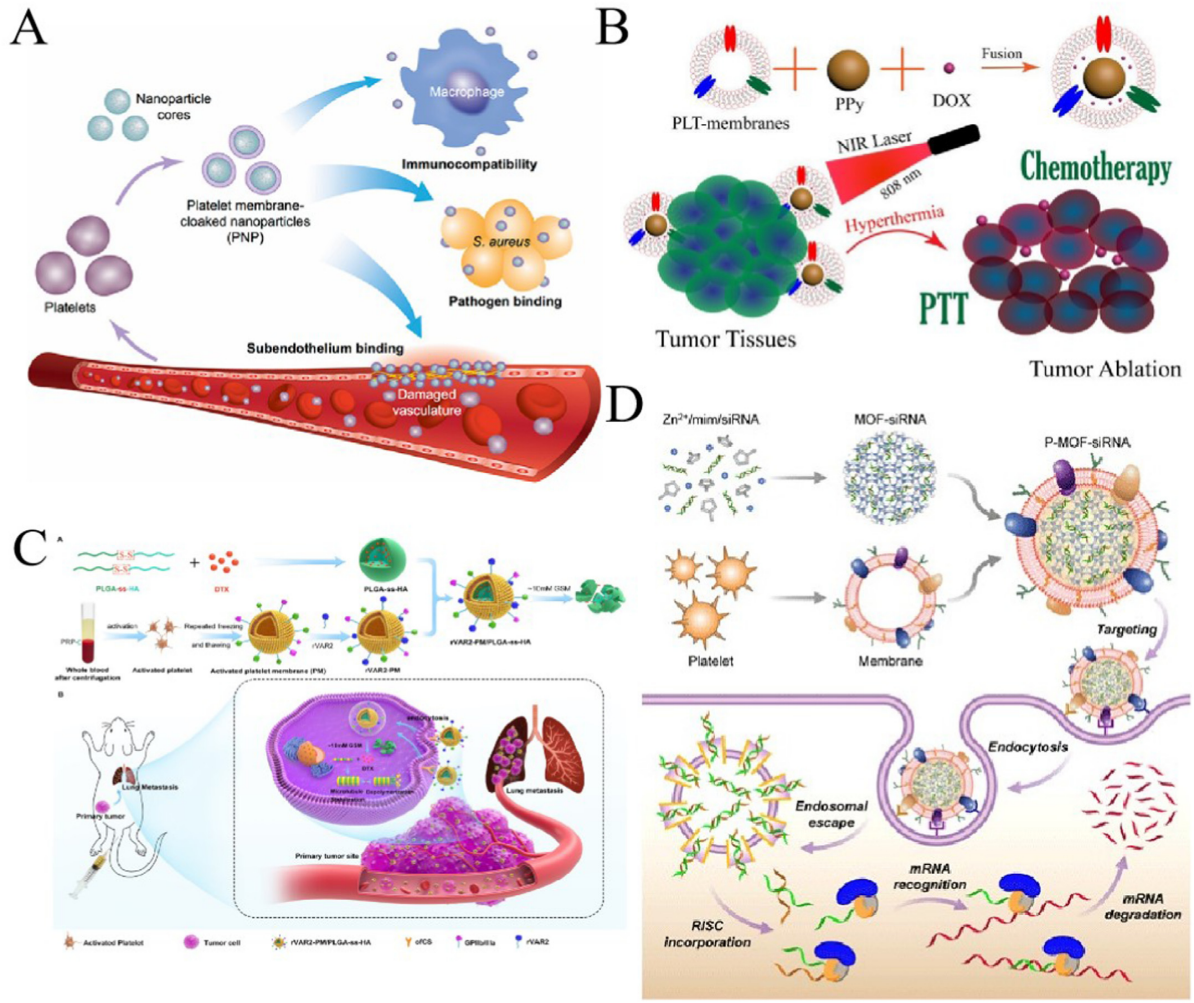
**Characteristics of platelets (PLTs) and application of PLT-membrane-coated nanoparticles (PLTMNPs) in tumor treatment. (A)** Characteristics of PLTMNPs; adapted and reproduced with permission from Springer Nature [108]. **(B)** PLTMNPs in photothermal- and chemotherapy; adapted and reproduced with permission from Royal Society of Chemistry [111]. **(C)** PLTMNPs prepared by modified PLTM for tumor chemotherapy; adapted and reproduced with permission from American Chemical Society [112]. **(D)** PLTMNPs in the cancer gene therapy; reproduced with permission from Creative Commons Attributes (CC BY NC) [113].

Platelets (PLTs) are small pieces of cytoplasm that are important for hemostasis in the body and have a long blood circulation time (average of 7–10 days) [107]. Owing to the presence of *p*-selectin and membrane proteins such as CD47, CD55, and CD59 on their surface, they can not only evade phagocytosis by *M*ø and prevent activation of the complement system but also recognize injured blood vessels as well as circulating tumor cells (CTCs) (Fig. 4A) [108]. These properties make PLTs ideal carriers to encapsulate drugs with wide potential for applications in oncology, cardiovascular disease, inflammation, and bacterial infections [109]. However, compared to other cell membranes, those of PLTs are smaller and have lower blood concentrations, which makes them more difficult to obtain, difficult to produce industrially, more individualized, and less stable [27,110].

There are many potential applications for PLT-membrane-coated NPs (PLTMNPs) in tumor therapy, and they have been widely studied. Wu et al. [111] packaged polypyrrole (PPy) and DOX into a PLTM (PLT-PPy-DOX) for photothermal and chemotherapy of hepatocellular carcinoma. The results showed that the PLT-PPy-DOX not only targeted tumors but also controlled the release of DOX during PTT (Fig. 4B). The recombinant VAR2CSA peptide (rVAR2) can specifically bind to chondroitin sulfonate on tumor cells. Zhou et al. [112] modified the PLT membrane with rVAR2 and encapsulated redox-reactive docetaxel-loaded NPs. The activated PLTM coated NPs showed good tumor-homing ability and controlled the release of chemotherapy drugs (Fig. 4C). Zhuang et al. [113] used PLTM is used to deliver siRNA, the PLTMNPs bound to target cells and were effective for cancer gene therapy (Fig. 4D). Another study used PLTMNPs to treat metastatic cervical cancer. P-selectin and CD47 on PLTM enable PLTMNPs to target subcutaneous and metastatic tumors, escape phagocytosis by Mø, effectively inhibit tumor invasion and metastasis, and avoid causing hematotoxicity [114]. These studies showed that PLTMNPs can not only avoid immune system clearance but also effectively target CTCs through their membrane surface, efficiently delivering anti-tumor drugs.

### 3.4 Stem cell membrane

It is well known that stem cells (SCs) are unspecialized cells, with self-renewal ability and differentiation abilities. Mesenchymal SCs (MSCs) are pluripotent and have all the common characteristics of SC including self-renewal and multidirectional differentiation ability and are able to circulate in the human body for a long time, have specificity for tumors, and are easily accessible with high immunomodulatory ability and low immunogenicity. At the same time, MSCs have a specific tumor-homing ability through chemokine receptor interactions and endothelial adhesion [115]. MSCs are easy to isolate and culture *in vitro*; however, the sources of MSCs are scarce, and obtaining MSCs requires considerable capital and medical resources. Bioengineering strategies combining synthetic NPs with SCs membranes, due to their tumor-targeting and -homing abilities [116], are now an ideal for preparing bionic carriers. Notably, SCs exhibit different growth effects in different tumor models [117], which means that the targeting ability of SCMNPs may be tumor specific and may not be applicable to all tumor types.

**Fig. 5:**
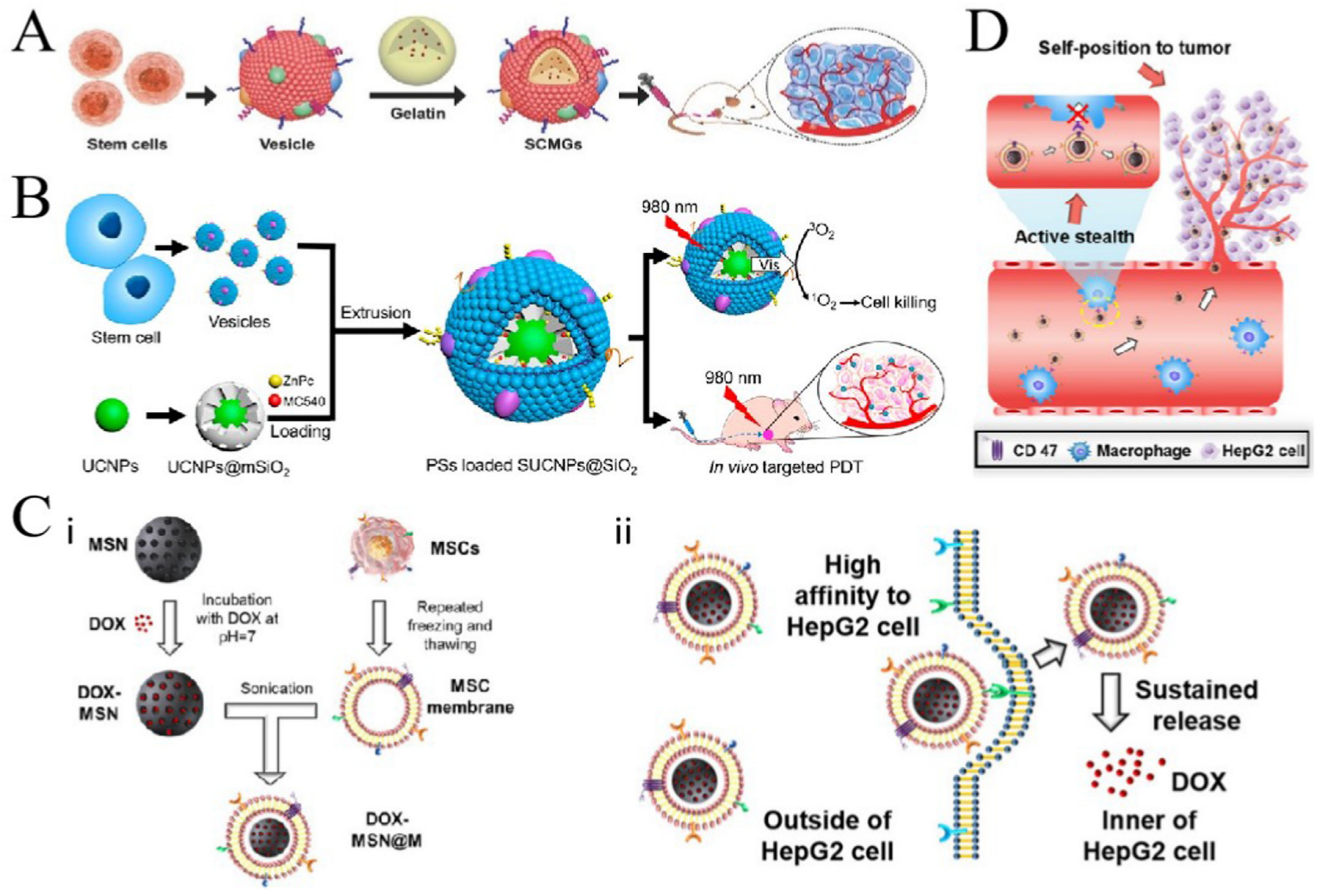
**Application of various stem cell membrane-coated nanoparticles (SCMNPs) in cancer therapy. (A)** Application of SCMNPs in chemotherapy; adapted and reproduced with permission from John Wiley and Sons [118]. **(B)** SCMNPs in photodynamic therapy of cancer; adapted and reproduced with permission from American Chemical Society [119]. **(C)** SCMNPs loaded with chemotherapy drugs showed high affinity to cancer cells; adapted and reproduced with permission from Elsevier [120]. **(D)** SCMNPs can evade the elimination by the immune system; adapted and reproduced with permission from Elsevier [120].

Animal studies have shown that SC membrane-coated nanogels containing DOX can accumulate around tumor lesions and effectively kill tumor cells without causing organ toxicity (Fig. 5A) [118]. Gao et al. [119] constructed a PDT system using bone marrow MSC (BMSC) membranes-coated with mesoporous silica (SiO2), UPNPs, and photosensitizers. The obtained SUCNPs@mSiO2 showed good biocompatibility and was used to treat cervical cancer. After intravenous injection, SUCNPs@mSiO2 showed a good immune escape ability, and the blood circulation time was significantly prolonged. SUCNPs@mSiO2 could effectively reach the tumor site, and the UPNPs activated photosensitizers for PDT, with a tumor growth inhibition effect of 66% (Fig. 5B). Li et al. [120] used MSCs membrane to encapsulate mesoporous silica nanoparticle (MSN@M). Compared to pure MSN, MSN@M was less frequently engulfed by Mø *in vitro*, showed a stronger tumor-targeting ability *in vivo*, and could load more than five times the amount of DOX. Their results showed that the DOX-loaded MSN@M reduced side effects in normal tissues due to its good targeting ability, effectively inhibiting tumor growth (Fig. 5C and D). Zhou et al. [121] prepared targetable NPs for oral squamous cell carcinoma by modifying the metal-organic backbone with dental pulp MSC membranes, and the results showed that the NPs were specific for oral squamous cell carcinoma, that adriamycin induced CAL27 ​cell death *in vitro* and blocked CAL27 tumor growth *in vivo*, and that the NPs were potential targeted drug delivery systems for oral squamous cell carcinoma.

### 3.5 White blood cell membranes

White blood cells (WBCs) play an important role in major diseases, like cancer and infections. Their powerful functions have inspired the development of WBC membrane-coated NPs (WBCMNPs). WBCMNPs have shown therapeutic potential because they inherit the entire source cell antigen profile, acting as a source cell decoy and mimicking the extensive biological interface characteristics of WBC membranes [122]. These include prolonged blood circulation, better cellular interactions, significant ability to recognize antigens for enhanced targeting, gradual drug release, and reduced *in vivo* toxicity [27]. The main WBCs used to encapsulate NPs are *M*ø, natural killer cells (NK cells), T cells, neutrophils (NEs), and dendritic cells (DCs).

**Fig. 6:**
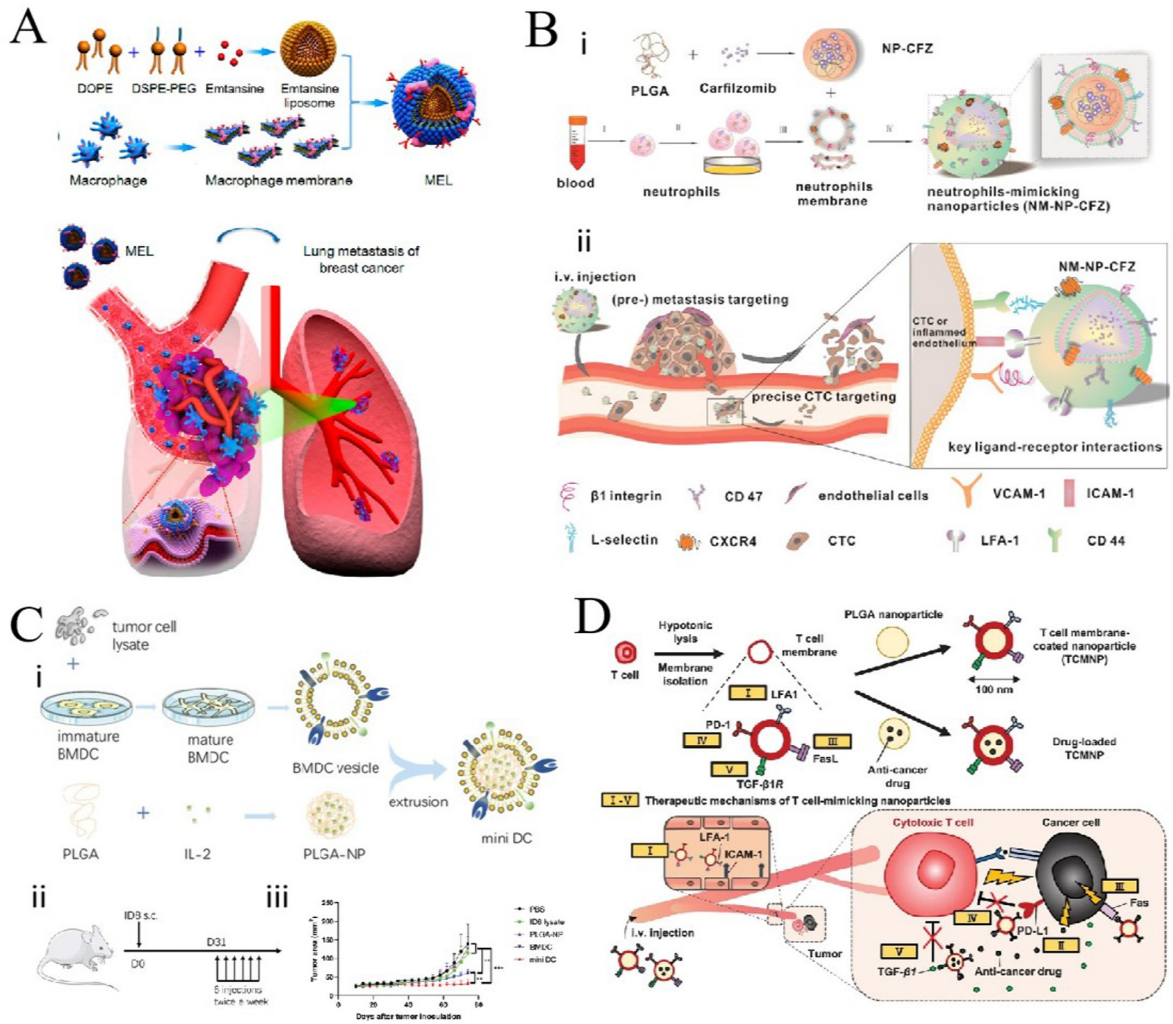
**Application of various white blood cell membrane-coated nanoparticles (WBCMNPs) in tumor treatments. (A)** Macrophage (Mø) coated NPs (Mø MNPs) in cancer therapy; adapted and reproduced with permission from American Chemical Society [42]. **(B)** Neutrophil (NE) coated NPs (NEMNPs) in the study of cancer therapy; adapted and reproduced with permission from American Chemical Society [137]. **(C)** Dendritic cell (DC) (DCMNPs) in the cancer therapy; reproduced with permission from Creative Commons Attributes (CC BY) [141]. **(D)** T cell coated NPs **(**TCMNPs) in the study of cancer therapy; adapted and reproduced with permission from John Wiley and Sons [144].

*M*ø play a key role in homeostasis, tissue repair, and immune response to pathogens, and they are also involved in inducing inflammatory responses and coordinating tissue repair [123,124]. The α4 integrin is a protein located on the surface of Mø, which can recognize the vascular cell adhesion molecule 1 (VCAM-1) on the surface of cancer cells, so it also has tumor-targeting ability [125]. Furthermore, this membrane masking can protect the core of NPs from the phagocytosis by immune cells, and it can neutralize endotoxin, increasing the safety of the therapy [126]. *M*ø are easy to obtain, culture, and purify. However, Mø are non-reproducing cells population that can live for 2–3 weeks under suitable conditions and are mostly used as primary cultures, making long–term survival difficult. Cao et al. [42] wrapped the drug–loading liposome with Mø membrane, which increased uptake by tumor cells and inhibited cell viability (Fig. 6A). Another study utilized pH-dependent *M*ø membrane-coated NPs (*M*øMNPs) that became unstable by sensing pH differences in the tumor microenvironment (TME), and thus slowly degraded to release antitumor drugs, and it has shown a strong therapeutic effect and targeting ability in human breast cancer cell lines [127]. Lai et al. [128] used *M*øMNPs to treat gliomas in situ. From their experimental results, the *M*øMNPs have good tumor-targeting ability and good therapeutic effects against glioma in situ.

NK cells are also important WBCs associated with antitumor, anti-infection, and immune regulation and can identify target cells. They are important in cancer treatment because they are able to defend against tumor cells expressing abnormal expression of cellular stress markers [129,130]. They can target tumor cells without previous antigen-specific-excitation and major histocompatibility complex (MHC), release cytotoxic perforin and granzymes, and secrete a series of cytokines, such as TNF- ⍺ [27]. NK cell membranes-coated NPs (NKCMNPs) showed tumor-homing ability and potential for targeted tumor therapy [131,132]. Meanwhile, NK cell immunotherapy is considered an effective cancer treatment and adjunct to standard cancer therapy [133]. For example, Deng et al. [134] used NKCMNPs for PTT. The results showed that the NKCMNPs were selectively accumulated in tumor and could induce or enhance the polarization of pro inflammatory M1 macrophages; they eliminated primary tumor growth and inhibited distant tumors.

NEs are the most abundant type of granulocyte, accounting for 40–70% of all WBCs in humans. They are the earliest WBCs to react to infectious and neoplastic inflammation, are closely related to the occurrence and development of cancer, and are excellent carriers of antitumor drugs [135,136]. The NEMNPs prepared by Kang et al. [137] is targeted to CTCs could effectively eliminate CTCs, and showed a good inhibitory effect against tumor metastasis (Fig. 6B). NE membranes could reduce immunogenicity after administration and allow the carrier to cross the blood-pancreatic barrier smoothly. Experiments in mice have shown that a NE membrane-coated ryanodine-poly (ethylene glycol)-poly (lactic acid-hydroxyacetic acid) copolymer is effective for the treatment of pancreatic cancer [138]. It also penetrates inflamed brain tumors, and NE membrane-NPs have been developed for the treatment of recurrent gliomas [139].

DCs are the strongest antigen-presenting cells named after the many dendritic or pseudopod-like protrusions that extend when they mature. The are the initial link in the initiation of the body's anti-tumor specific immune response and plays an important role in activating innate and adaptive immune responses. DCs can load various types of antigen substances, including nucleic acids, polypeptides, and protein antigens and induce specific immune responses [140]. Cheng et al. [141] developed a bionic nanovaccine derived from DCs. They extracted a membrane of ovarian cancer cell lysate-primed DCs and used it to wrap the PLGA loaded with IL-2, which they named “miniDC”. This inherited the antigen profile and stimulated enhanced T-cell activation both *in vitro* and *in vivo*. The “MiniDC” showed good tumor treatment and defense abilities for ovarian cancer in their animal experiments (Fig. 6C).

T cells, derived from the bone marrow, are also important WBCs in the body. T cells can migrate to tumor sites and recognize antigens on the tumor surface, thus activating antitumor immune responses and playing a key role in tumor immune surveillance [142,143]. Kang et al. [144] used T-cell membrane-coated PLGA that loaded with the anticancer drug dacarbazine for the treatment of melanoma. The TCMNPs can target and tumors through T cell membrane-derived proteins, release anticancer drugs, and restore depleted CTL, thereby killing cancer cells (Fig. 6D). TCMNPs had higher therapeutic efficacy than immune checkpoint blockade in the treatment of melanoma. Meanwhile, the use of immunotherapy regimens using T-cell membrane-encapsulated nanomaterials instead of commonly used immunotherapy has been increasing in recent years [145].

### 3.6 Other membranes

In addition to cell membranes from the humans, bacterial membrane-coated NPs have also been studied. Bacterial membranes contain a large number of immunogenic antigens, so they are the preferred materials for vaccinations [146]. Simultaneously, bacterial membranes play a key role in promoting adaptive immune responses and stimulating innate immunity. Zhang et al. [147] collected bacterial outer membrane vesicles and used them to coat Au NPs, which also induced elevated interferon-γ and interleukin-17 levels, showing the antibacterial properties of the NPs. However, relatively few bacterial membrane-encapsulated nanodrugs have been used in cancer research. Patel et al. [148] developed a bacterial membrane-coated NPs (BNP), which was consisted with immune-activated PC7A/CpG multimeric nuclei encapsulated in bacterial membranes and imide moieties to enhance antigen delivery. In mice with homologous melanoma or neuroblastoma, the BNP combined with radio- and immunotherapy showed that new tumor antigens were captured after radiation treatment, the uptake in DCs was enhanced, and the anti-tumor T cell response was also stimulated by the cross presentation [148].

**Fig. 7:**
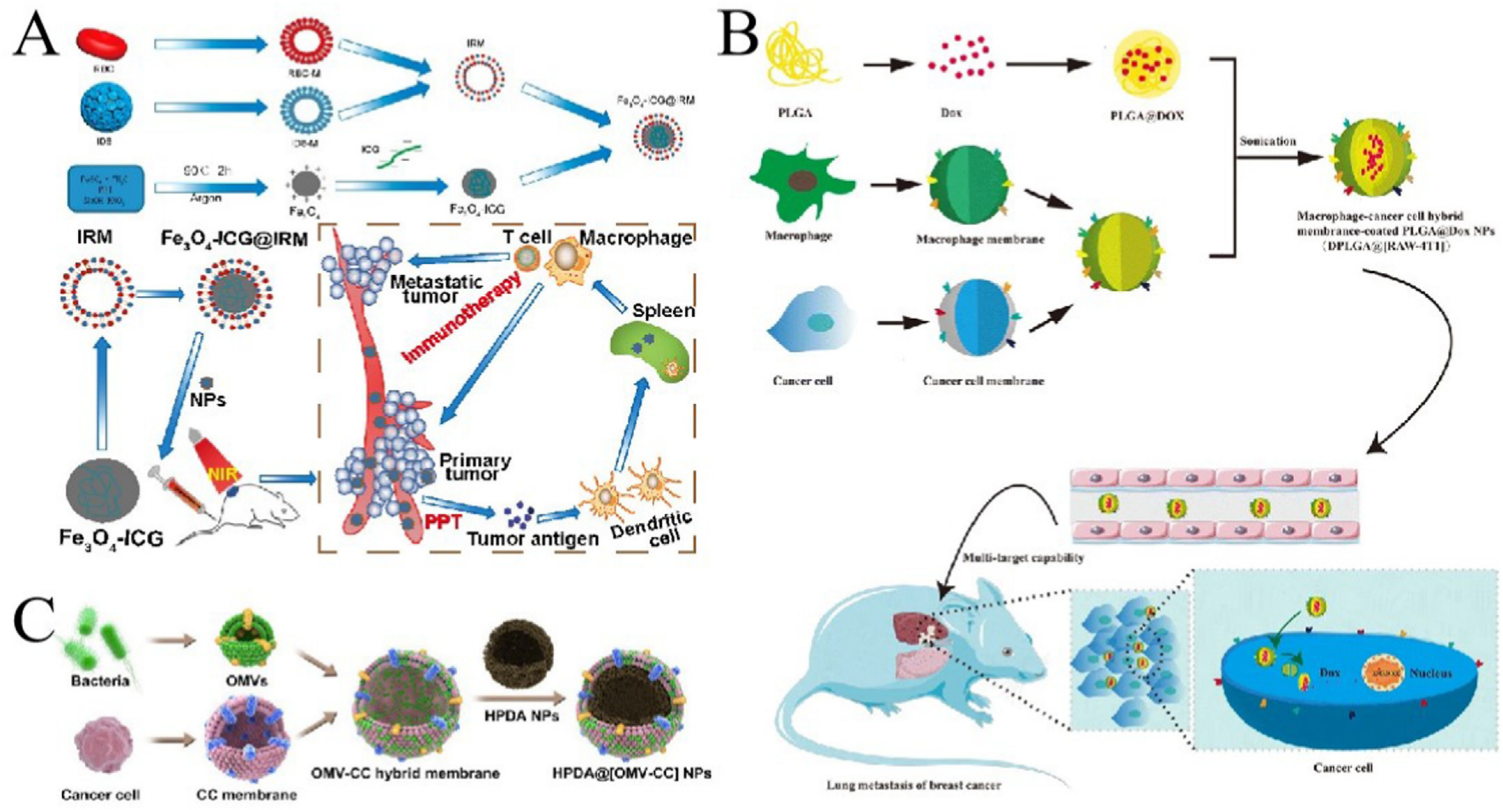
**Application of hybrid membrane-coated nanoparticles (HMNPs) in cancer treatments. (A)** Macrophage-cancer hybrid membrane-coated nanoparticles; adapted and reproduced with permission from American Chemical Society [156]. **(B)** Macrophage-cancer hybrid membrane-coated nanoparticles; reproduced with permission from Creative Commons Attributes (CC BY 4.0) [157]. **(C)**. Bacterial Vesicle-Cancer Cell Hybrid Membrane-coated Nanoparticles; adapted and reproduced with permission from American Chemical Society [158].

In addition the use of single-cell membranes as envelope membranes, hybrid cell membranes have been increasingly investigated in recent years. Hybrid cell membrane-coated NPs retain the physicochemical properties of NPs and inherit the biological functions of the source cells and also confer multiple biological functions derived from the original cells [149]. Since biological functions are derived from the cell membrane, the criteria for selecting cell membranes depend mainly on the unique characteristics of the source cells and the requirements of cancer therapy. The RBC-PLT hybrid membrane was the first hybrid bionic cell membrane studied. Zhang et al. [150] reported in 2017 a hybrid RBC-PLT membrane-camouflaged NPs with surface-labeled membrane proteins from both cells, and the hybrid membrane-coated NPs showed the cross characteristics of single film-coated NPs. Subsequently, researchers successfully prepared RBC-cancer hybrid cell membranes-coated NPs [151,152], PLT-WBC hybrid cell membrane coated NPs [153] cancer stem cell-Platelet hybrid cell membrane-coated NPs [154], cancer cell-PLT hybrid CMNPs [155], and cancer cell-RBC hybrid cell membrane-coated NPs (Fig. 7A) [156] for personalized cancer treatment and other applications. Mø-cancer hybrid membranes showed good targeting and effectively inhibited lung metastasis of breast cancer (Fig. 7B) [157]. Wu et al. [155] evaluated the targeting and anticancer activities of PLTs and cancer cell membranes-camouflaged lipid NPs loaded with β-mangostins. The cell and animal experiments demonstrated their is efficacy in glioma chemotherapy. Furthermore, Wang et al. [158] prepared a hybrid membrane from bacterial and cancer cell membranes, which showed nano DDS targeting and immune activation abilities (Fig. 7C).

The modified cell membrane is also a special type of cell membrane because the function of each type of cell membrane is different, For example, the RBCMs has a relatively low targeting ability [24], but modifying the cell membrane can diversify the function of CMNPs. There are many ways to modify the cell membranes. The first is to add functional substances to the cell membrane, such as chemicals and proteins. By binding DSPE-PEG-FA molecules onto RBCMs. Li et al. [103] obtained synthesized RBCMNPs with stronger targeting ability. Zhou et al. [112] enhanced the tumor-homing ability of PLTMNPs by modifying the PLT membrane with recombinant VAR2CSA peptide, thereby enhancing the bioavailability of the drugs. In addition to increasing some properties of cell membrane, modifications can also increase the therapeutic effect of NPs. Zhao et al. [159] proposed the concept of antibody vesicles, which is a new technology to modify antibodies on cell membrane vesicles. This method improves the targeting ability and stability of cell membranes and can increase the therapeutic effect due to the biological characteristics of the antibody. This technology includes three main methods: chemical conjugation, genetic engineering, and membrane hybridization. For example, Zhang et al. [160] genetically engineered megakaryocytes and then obtained PLTs expressing programmed cell death protein 1 (PD-1), which increased the targeting ability of membrane vesicles in immunotherapy. A similar effect can be obtained by modifying the cell membrane on CMNPs with antibodies. Lang et al. [161] modified a PLT-leucocyte hybrid membrane with special antibodies and used it to coat immunomagnetic beads. The HM-IMBs can effectively isolate CTCs. Another way is to modify the cell membrane at the gene level. For example, Zhang et al. [162] genetically modified a source cell line to make it express very late antigen-4 (VLA-4) on the cell membrane. CMNPs prepared with the engineered membrane showed a stronger affinity for target cells. Moreover, Qing et al. [163]. developed a general and cell-friendly supramolecular strategy to engineer cell membrane vesicles. This supramolecular engineering methodology is based on non-equivalent interactions, which avoids potential damage to cell membrane function and provides a new idea for cell membrane modification of nanomaterials.

Liposomes are naturally found in cell membranes and are composed of the same components as the membranes. In addition to serving as the core of nano drug carriers as described previously, liposomes can also serve as a membrane to wrap the nano core. They have many advantages such as good biocompatibility, non-immunogenicity, and easy surface functionalization, and are the most studied and widely used nanodrug carriers [164]. Liposomes themselves have a certain passive targeting ability based on the EPR effect, and the enrichment rate of liposome-encapsulated drugs alone at tumor sites remains low. Moreover, liposomes are highly modifiable. For example, Zhang et al. [43] used modified liposomes and cancer cell membranes to prepare hybrid membranes to coat NPs for precise treatment of non-small cell lung cancer.

## 4 Generation and processing of cell membrane coated NPs

**Fig. 8:**
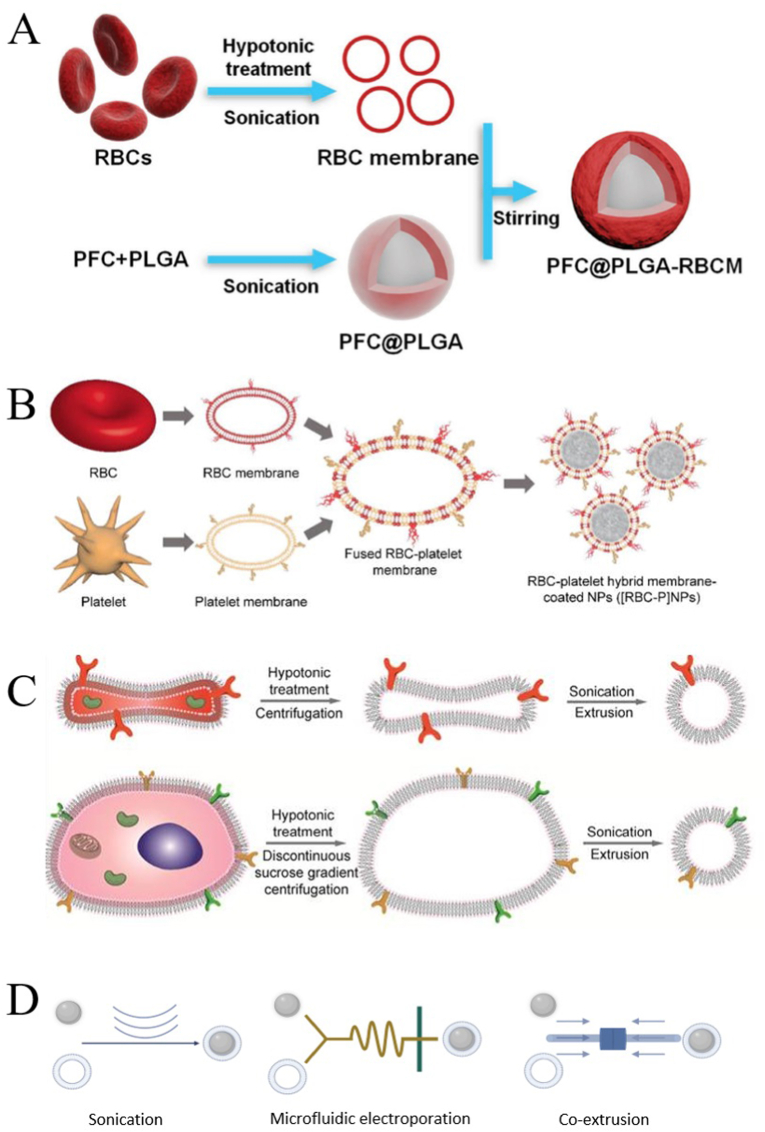
**Synthesis process of the cell membrane-coated nanoparticles (CMNPs). (A)** NPs wrapped in single cell membrane; adapted and reproduced with permission from John Wiley and Sons [199]. **(B)** NPs wrapped in a hybrid membrane; adapted and reproduced with permission from John Wiley and Sons [150]. **(C)** Comparison of membrane vesicles formed by nucleus-free cells (top) and eukaryotic cells (bottom); reproduced with permission from Creative Commons Attributes (CC BY NC) [195]. **(D)** Three ways of integrating of NPs and cell membranes (Created using BioRender.com.).

The preparation process of CMNPs is usually divided into three steps: the extraction of the cell membrane, the preparation of the NPs core, and the assembly of the cell membrane shell and NPs core. Fig. 8A and B shows the process of wrapping NPs in a single cell membrane and hybrid membrane, respectively. We list the main technologies and techniques involved in creating CMNPs below.

### 4.1 Extraction of cell membrane

Zhang et al. [100] first proposed the technique of RBCM-coated NPs in 2011, and this pioneering study of NP functionalization opened a more unique pathway, bypassing the complex surface molecular identification, purification, and binding processes, transferred the function of RBCMs intact, and successfully prepared long-cycle NPs. However, the methods for extracting cell membranes from different sources and types are roughly the same and are mainly divided into two categories. The chemical or mild cracking method includes hypotonic treatment and chemical lysis and temperature treatment (repeated freezing and thawing). This type of extraction method is relatively mild and does not result in mechanical damage to cells. The other method is physical and comparatively damaging because it includes mechanical destruction, differential centrifugation, pressure homogenization, and crushing with a mortal and pestle [27,[191], [192], [193], [194]]. The choice of method mainly depends on the type of cell membrane and the location and properties of the membrane protein. Hypotonic treatment or repeated freeze-thaw is recommended for RBC and PLTs because no complicated operation is required. Mechanical lysis or combined with hypotonic treatment is usually recommended for larger cells. The degree of cell lysis using this method is higher, but it may damage the cell membrane [27,195]. Moreover, the process foe extracting cell membranes slightly differs between nucleated cells and nucleus-free cells. For nucleus-free cells (Fig. 8C), such as RBC and PLTs, sufficient RBCs and PLTs are separated from whole blood and PLT-rich plasma, respectively, and then cells are lysed by hypotonic treatment or repeated freezing and thawing, and finally cell membranes isolated [108,196]. For eukaryotic cells such as cancer cells and T cells (Fig. 8C), the steps for extracting cell membranes are more complex. First, since the source cells may not be sufficient for direct use after separation *in vivo*, enrichment and purification are required [42]. In addition to swelling the primary cells or cell lines in a hypotonic solution, mechanical lysis is required. For example, the cells are crushed or squeezed by ultrasound, the cell membrane is broken and homogenized, and then the components inside the cells are removed by gradient centrifugation to separate the cell membrane [197]. Eukaryotic cells contain nuclear membrane in addition to the outer membrane, so specific markers are used to detect the outer membrane and nuclear membranes to determine whether the extraction of the cell membrane was successful. For example, for *M*ø, F4/80, toll like receptor-4, CD206, and CD11c are markers of the cell membranes, whereas lamin A/C and receptor interacting protein 140 are markers of the nuclei. The purity of cell membranes can be determined by detecting the expression of the corresponding markers on the extracted membranes [26].

In addition to the simple modification of the cell membrane on NPs, hybrid membranes have also been studied in recent years to combine the advantages of different membranes. Fig. 8B is a schematic diagram of hybrid membranes preparation. Hybrid membranes are prepared through the membrane hydration method. After obtaining the two kinds of cell membranes in the above way, they are stirred at 37 ​°C and placed in an ice bath or sonication. Then two membranes are fused together [150]. Another method involves fusing two different cells together to form a fused cell that retains the surface features of the source cells, and then extracting the cell membrane using the above method. However, the fused cells obtained by this method may have the same origin; therefore, it is necessary to screen the fused cells [198].

### 4.2 Integration of NPs and cell membranes

Methods commonly used to wrap NPs in cell membranes include mechanical extrusion, ultrasound, and microcurrent perforation (Fig. 8D).

#### 4.2.1 Co-extrusion

Mechanical extrusion is the original method used for cell membrane wrapping of NPs, in which the NP core and purified cell membrane shell are co-extruded through a porous membrane [100]. The mechanical forces generated by extrusion enable reorganization around the core NPs by disrupting the cell membrane structure. Owing to the fluidity of the cell membrane, the mechanical force in the extrusion process makes NPs pass through the phospholipid bilayer and helps the cell membrane to wrap NPs to synthesize CMNPs [32]. As the diameter of the extrusion film hole is consistent, the size of the extruded CNMPs can be guaranteed to be consistent, and the extrusion process does not involve chemical reactions, so the protein on the cell membrane are largely retained to ensure the activity of the cell membrane [200]. However, co-extrusion commonly causes waste of raw materials, because it is easy to leave materials on the extrusion film.

#### 4.2.2 Sonication

Ultrasonication has emerged as a new method in recent years, and cell membranes can be destroyed through ultrasonic energy. NPs and cell membrane fragments can form CMNPs through non-covalent interactions, and the surface charge of cell membranes and the electrical properties of NPs can also help the synthesis of CNMPs [149]. Cell membranes and NPs can spontaneously form core-shell nanostructures. This method consumes less materials, and damage to proteins on the cell membrane can be avoided by adjusting parameters. However, the size of CMNPs may be different, the cell membrane coating may be uneven, and the integrity of the wrapping is poor.

#### 4.2.3 Microfluidic electroporation

The microfluidic electroporation method has been a newly developed as an amplification method in recent years. It has been successfully used to wrap magnetic NPs in RBC films [201]. The device consists of a Y-shaped merging channel, S-shaped mixing channel, and electroporation area in front of the outlet. The key to making CMNPs is pulse duration, fine-tuning the pulse voltage, and flow rate. This method has great advantages in reducing membrane protein damage and raw material loss, but the technical cost is relatively high [197].

#### 4.2.4 Other encapsulation methods

Other encapsulation methods, such as specific binding, refer to finding receptors on the cell membrane, connecting the corresponding ligands with NPs, and combining them using the interaction between receptors and ligands. However, this method reduces the protective effect of NPs on the cell membrane and is limited in practical applications [202]. Zhang et al. [203] synthesized cell membrane-coated hydrogel NPs through in site polymerization. The in site polymerization, the principle of this method is to expose the reaction mixture to a heat source or radiation source to initiate polymerization [204].

### 4.3 Characteristic analysis of CMNPs

**Fig. 9:**
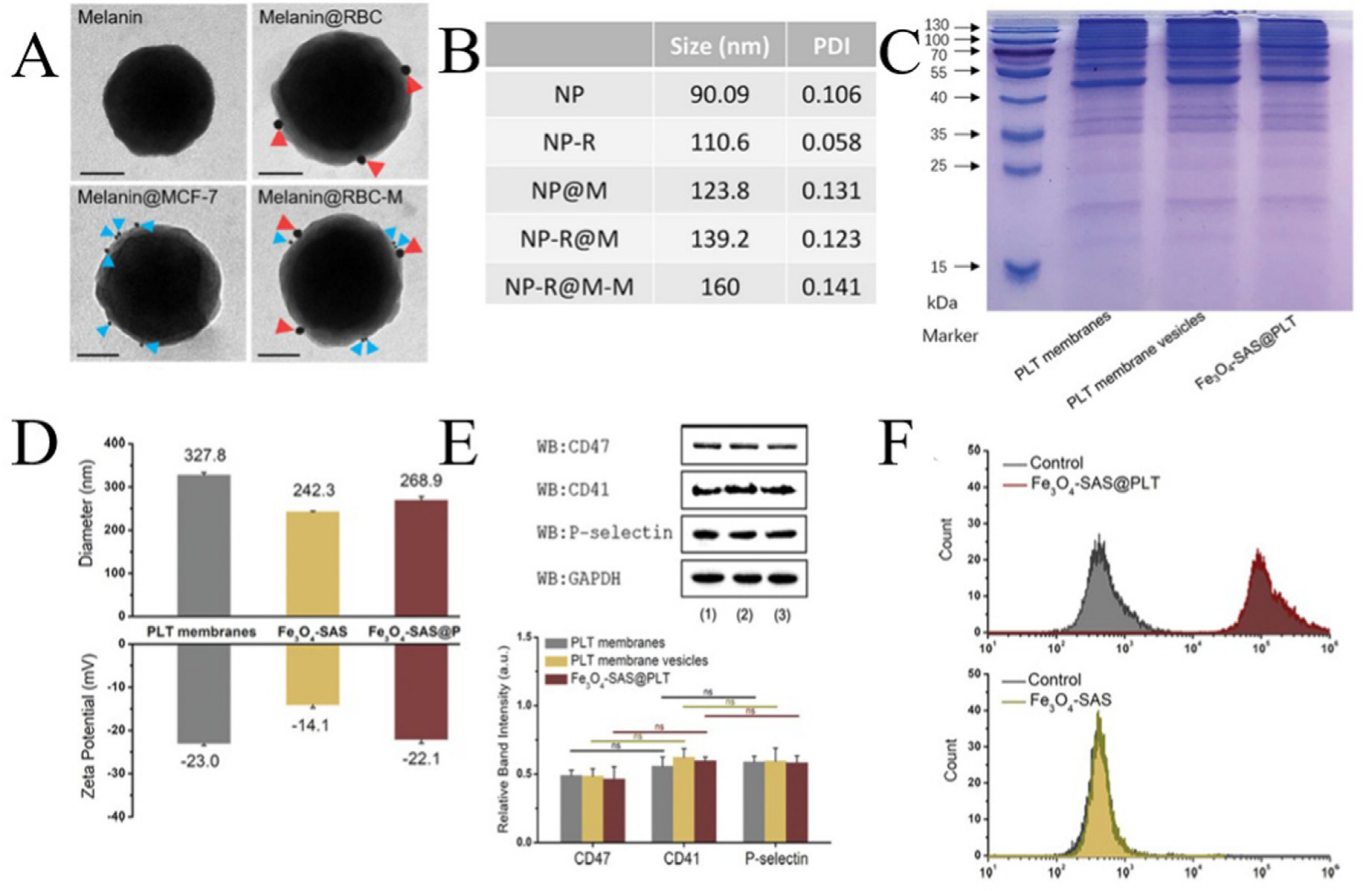
**Characterization of cell-membrane coated nanoparticles (CMNPs). (A)** The immunogold transmission electron micrographs: bare NPs, RBCMNPs, CCMNPs, HCMNPs adapted and reproduced with permission from Elsevier [152]. **(B)** Dynamic light scattering (DLS); adapted and reproduced with permission from American Chemical Society [94]. **(C)** Analysis of protein composition by sodium dodecyl sulfate-polyacrylamide gel electrophoresis [177]. **(D)** Hydrodynamic diameter and zeta potential [177]. **(E)** Western blotting for protein analysis [177]. **(F)** Measurement of protein activity by flow cytometry; adapted and reproduced with permission from John Wiley and Sons [177].

When the shell structure of the cell membrane and NPs is perfect, certain technologies are needed to determine whether the biofilm and NPs are successfully loaded. First, the integrity of the capsule can be observed by microscopic analysis and glycoprotein and sialic acid detection. Then, the biological characteristics can be detected by gel electrophoresis, immunoblotting, immunohistochemistry, immunofluorescence, ELISA, and UV visible absorption spectroscopy to determine whether the biofilm and NPs are successfully loaded. Fig. 9(A-E) shows some characteristic analyses of CMNPs. Fig. 9A shows the morphology of bare NPs, RBCMNPs, CCMNPs, HCMNPs under immunogold transmission electron microscopy, positive results of two fluorescent markers showed that the hybrid membrane successfully encapsulated NPs. Fig. 9B shows the results of dynamic light scattering. The CMNPs(NP-R@M) are large after being coated with the cell membrane compared with NP-R, and the diameter is larger after modification with mannose moiety (NP-R@M-M). The protein composition of CMNPs was similar to that of the cell membrane as indicated through sodium dodecyl sulfate-polyacrylamide gel electrophoresis (SDS-PAGE) (Fig. 9C). Fig. 9D shows that the hydrodynamic diameters of CMNPs are larger than those of naked NPs, and the zeta potentials of CMNPs were similar to those of cell membrane vesicles and lower than those of naked NPs. Western blotting showed that the specific protein markers on the cell membrane are effectively retained on CMNPs (Fig. 9E). Analysis of protein activity on CMNPs by flow cytometry (Fig. 9E) showed that functional proteins were successfully integrated onto CMNPs.

## 5 Characteristics and advantages of CMNPs in tumor therapy

The nanodrug delivery system (nanoDDS) has many unique advantages in drug delivery, such as highly adjustable physicochemical properties, but it also has some limitations, including being captured and cleared by the immune system, low biocompatibility, and potential toxicity. Thus, there are considerable obstacles to applying nanoDDS [205]. Exogenous nanodrugs without surface modification will be swallowed by the *M*ø of the reticuloendothelial system, which clears most of the nanodrugs in the liver and spleen, reducing circulation and homing to the target site well [88,206]. Liposomes have proven to be an effective NanoDDS [207]. However, although it has a phospholipid bilayer structure similar to that of natural cells, instability caused by the lack of a complete membrane structure is still a major limitation for its use as a nanoDDS [208]. The cell membrane is used to encapsulate and coat the nanodrug loading system, which integrates the advantages of the nanodrug loading system, such as high drug loading capacity and good biocompatibility of cellular carriers, and discards their respective disadvantages as carriers, thus significantly improving the stability of NPs and reducing drug leakage [88,200]. Through rational design of drug carriers, drugs can be delivered to the target in two modes, passive or active targeting, to increase drug aggregation at the target location, and using cell membranes to modify NPs is a good way to accomplish targeting. Because the extracted cell membrane protein remains on its surface, it has inherent characteristics of its mother cell. Therefore, the cell membrane can provide favorable biological functions for coated NPs, and the corresponding advantages of cells from different sources are also different.

### 5.1 Good biocompatibility

Most nanoDDSs mainly focus on synthesis strategies, requiring lengthy synthesis and optimization, especially when integrating multiple functional forms into a single NP. However, the biocompatibility of synthetic materials is unsatisfactory. For example, PEG, as a nanodrug carrier, has also been observed to accelerate blood clearance, although PEG was previously considered inert [209,210]. Therefore, NPs with adjustable surface properties and good biocompatibility are required. As nano carriers, cell membranes can maintain good biocompatibility from the source cells, have relatively low cytotoxicity and immunogenicity, and reduce drug aggregation in internal organs.

### 5.2 Immune escape and long cycle time

Some factors on the cell membrane can release the a “Don't eat me” signal, leading to escape from the immune system of the body. This effectively extends the cycle time. For example, CD47 on the RBC surface selectively interacts with SIRP-α expressed by *M*ø to escape *M*ø e uptake. In addition, the life of RBCs can be as long as 120 days, resulting in a longer cycle time [211]. Like RBC, PLTs also contain CD47 on their membranes. At the same time, PLTs contain unique surface parts that promote adhesion under the endothelium and pathogen interaction, and can avoid phagocytosis by *M*ø [212,213]. In addition, there are other proteins on the PLT membrane, such as CD55 and CD59, which can inhibit the complement system [108,174]. Some WBCs in the human body are also mainly in tissues and blood. NPs encapsulated by *M*ø/monocytes or NE membranes delay the uptake of phagocytes by reducing conditioning and self-recognition mechanisms. At the same time, cytotoxic T cells can normally circulate in the body in search of antigens, while NKCs provide host defense. Therefore, NPs modified by WBC membranes can also be effectively circulated in the human body [122]. In addition, CCMNPs have a long cycle time because tumor progression and metastasis are mainly due to immune tolerance of the body to malignant cells. During the development of cancer, cancer cells developed complex mechanisms to escape the attack from the body's immune system, which plays an important role in immune escape through overexpression of CD47 molecules [214].

### 5.3 Disease targeting

Traditional nanodrug carriers play an extremely important role in pharmaceutical applications. Nanodrug carriers made of natural or synthetic polymer-like compounds have low storage at target locations, low drug utilization, and even have certain toxicity to the body. The modification of NPs by the cell membrane can improve the targeting effect of diseases to a certain extent. PLTs usually accumulate in damaged tissues and trigger the repair process. In addition, surface molecules such as P-selectin and CD40, ligand on the PLT membrane, participate in the regulation of disease processes, especially inflammatory events and cancers [215,216]. For example, P-selectin binds to CD44 molecules, which are expressed on the surface of tumor cells, and PLTMNPs have been shown to target and accumulate in tumor tissues [174].

WBC themselves are part of the human immune system and so are usually not captured by the immune system and can cross the body's biological barrier to reach the target tissue. WBC targeting ability depends mainly on the lymphocyte function-related antigen-1 (LFA-1) molecule, which can bind to intercellular adhesion molecule-1 (ICAM-1) on inflammatory endothelial cells [217]. The monocyte/*M*ø system includes monocytes in the blood and inflammatory cell drug carriers, which have been studied most frequently, or migratory Mø. As early as 2007, Choi et al. [218] found that α-integrin is connected with VCAM-1 on tumor cells, which results in the high efficiency of *M*ø as a transport drug that acts like a “Trojan Horse” with tumor cells. Moreover, there is a special membrane protein on the surface of monocytes and *M*ø that can be recruited to the tumor site by C–C chemokine ligand 2 [219]. NEs can bind to circulating tumor cells or inflammatory endothelial cells through ligand receptor interactions, such as CD44 and L-selectin, LFA-1 and ICAM-1, and β 1 integrin and VCAM-1 [137]. The expression level of adhesion molecules of cytotoxic T lymphocytes is higher; therefore, it has a better tumor-targeting ability [220]. NKCs target cancer cells by inhibiting and activating receptor proteins on their surfaces (such as NKG2D and DNAM-1) [221].

CTCs originate from the original tumor. They can escape immune surveillance and target the same types of tumors through cell surface interactions, including *E*-cadherin and Thomsen–Friedenreich antigen. Furthermore, homotypic cancer cell aggregations are an important basis for distant metastasis of cancer [222]. This also provides a new possibility for CCMNPs to actively target tumors and track and capture cancer cells in the blood.

In addition, SCs adhere to LFA-1 and ICAM-1, making them suitable for cellular drug delivery [223]. However, the targeting ability of RBCs is relatively low. As described in Section 3.6, in order to enhance the targeting ability of RBCs, they can be modified by inserting ligands that specifically bind to inflammatory tissues or tumor cells onto the RBCM.

### 5.4 Other characteristics

CMNPs have a high drug-loading capacity. Some NPs themselves have a high drug-loading capacity, and CMNPs have a good targeting ability and can release drugs in the tumor to avoid drug loss in circulation. Xie et al. [224] coated RBCM onto the porous PLGA loaded with curcumin. The system had extremely low toxicity, high encapsulation rate (>93.8%) and drug loading (>8%), and can control the release of drugs, achieving good anti-cancer effect.

In addition, the ability to cross biological barriers such as the blood-brain barrier is an important advantage of CMNPs, because most therapeutic drugs cannot cross these biological barriers. Even though some NPs can cross the blood-brain barrier, the low drug loading and poor targeting ability make it difficult for drugs to be effective in the brain [225]. By inheriting the biological characteristics of the source cell through membrane modification, it is possible for therapeutic drugs to cross these barriers and avoid being eliminated by the immune system. A recent study showed that RBCMs can successfully target the transferrin receptor at the blood-brain barrier and glioma surface, as well as CD13 that is highly expressed by tumor cells, through the dual modification of T7 peptide and polypeptide NGR, and significantly enhance the anti-glioma effect [226]. Furthermore, the blood-pancreas barrier is a challenge for the treatment of pancreatic cancer because drugs have difficulty passing through this barrier to enter the pancreas. In the study of Cao et al. [138], they used NE membranes to coat a PLGA loaded with celastrol. The CMNP overcame the blood–pancreas barrier and allowed the drugs to selectively accumulate in the tumor. This demonstrated specific pancreatic drug delivery and prolonged the life of mice by minimizing liver metastasis of tumors.

## 6 Current status of CMNPs in tumor therapy

### 6.1 Targeted chemotherapy of tumor

Chemotherapy is one of the most widely used treatments methods for malignant tumors. The biggest disadvantage of chemotherapy drugs is that they attack cells indiscriminately and lead to characteristic side effects such as hair loss, vomiting, and bone marrow suppression. Therefore, improving the targeting of chemotherapy drugs is necessary to reduce the damage to normal cells. Targeted chemotherapy can be achieved by combining CMNPs that with chemotherapy drugs and utilizing the targeting characteristics of the cell membrane.

**Fig. 10:**
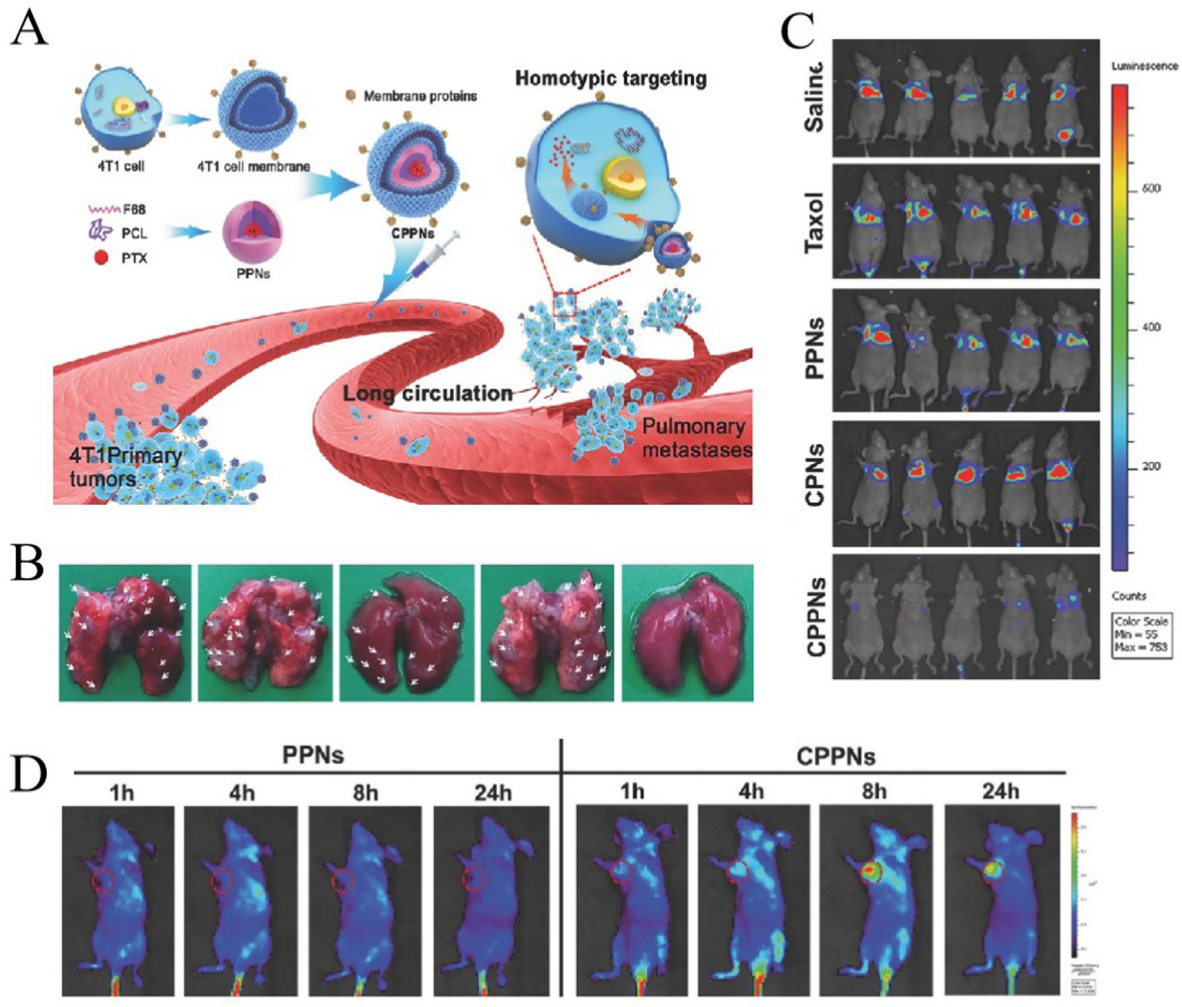
**Application of cell membrane-coated nanoparticles (CMNPs) in cancer chemotherapy**; adapted and reproduced with permission from John Wiley and Sons [227]**. (A)** Synthesis of CCMNPs and its application in targeted chemotherapy of lung metastasis of the breast cancer cells. From left to right are saline, paclitaxel, paclitaxel loaded polymeric NPs, cell membrane coated polymeric NPs, and CPPNs. **(B)** Number of pulmonary metastatic nodules after different NPs treatments. **(C)***In vivo* bioluminescence imaging of lung metastasis of breast cancer in mice. **(D)** Images of different NPs stacking in lung metastasis model of breast cancer Mice. PPNs, prepared polymer NPs.

Sun et al. [227] developed a strategy to target homotype tumors using NPs wrapped in cancer cell membranes. They extracted cancer cell membranes from 4T1 breast cells, prepared polymer NPs (PPNs) loaded with paclitaxel from polycaprolactone and copolymer F68, and finally formed nanodrugs (CPPNs) wrapped in cancer cell membranes, which have high targeting specificity for the same type of cancer cells (Fig. 10A). As depicted in Fig. 10B, at the end of the experiment, the metastatic foci in lung tissue after CPPN treatment were significantly fewer (on the far right), which shows that CPPN further improved the anti-metastasis effect of PPN. As illustrated in Fig. 10C, the *in vivo* bioluminescence imaging of lung metastasis of breast cancer also showed the anti-metastasis ability of CPPNs. As shown in Fig. 10D, fluorescence imaging showed the distribution of CPPNs and PPNs in mice at different time points. The accumulation of CPPN in tumor is increased in the first 8 ​h, and the tumor aggregation of CPPN was better than that of PPN, demonstrating the targeting ability of CPPNs.

### 6.2 Photothermal and photodynamic therapy

For PTT of tumors, the photothermal agent is irradiated by light at a specific wavelength to increase its temperature and kill the tumor cells, For PDT, photosensitizers can produce many ROS under specific light irradiation, which can kill tumor cells. For PTT and PDT, it is important to target drugs to tumors and avoid being cleared by the immune system [228]. Given the advantages of cell membranes in terms targeting and biocompatibility of cell membranes, photothermal agents, photosensitizers, and other drugs can be targeted and efficiently transported to the tumor site by CMNPs.

**Fig. 11:**
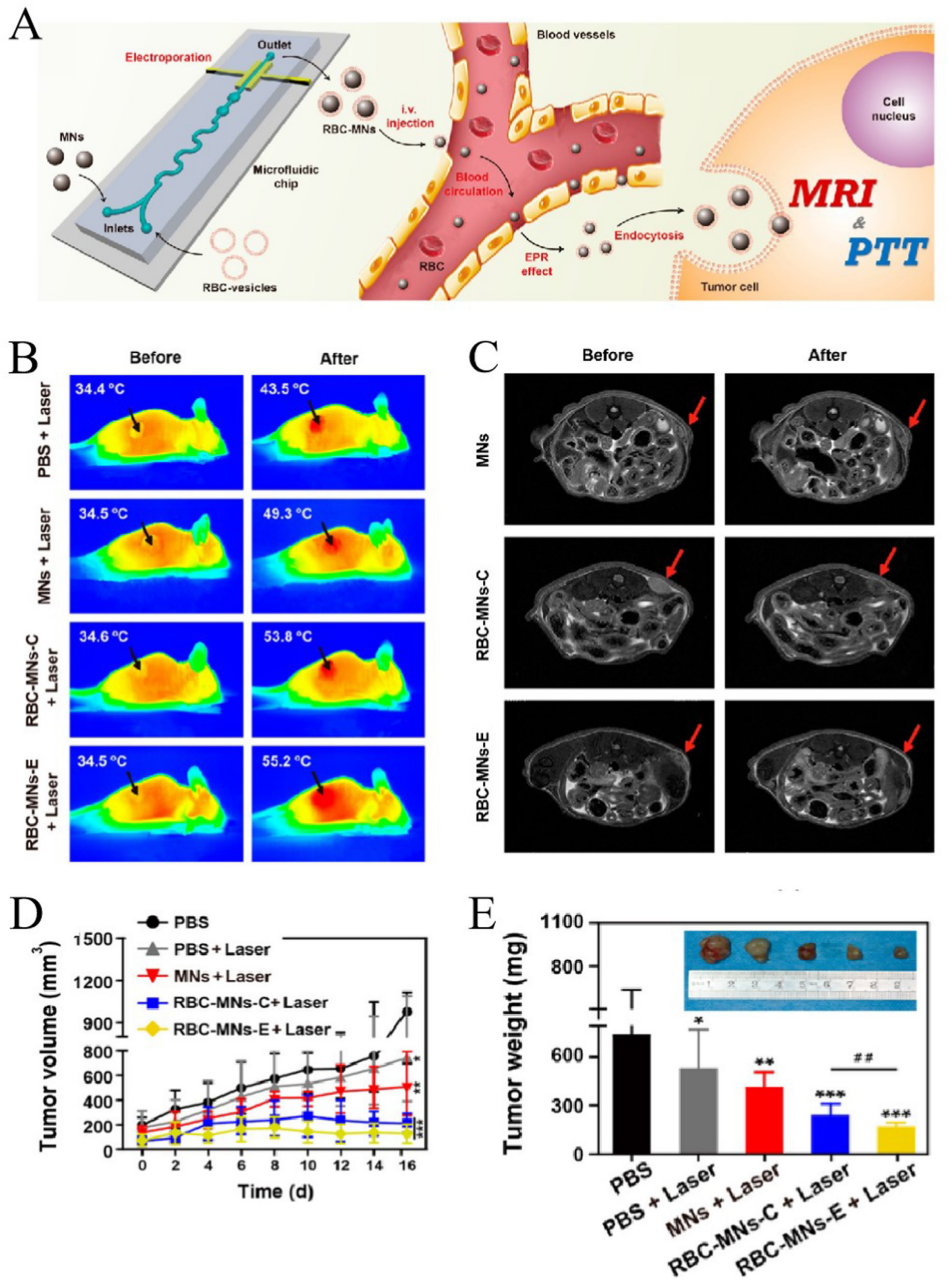
**Application of cell membrane-coated nanoparticles (CMNPs) in photothermal therapy and imaging of cancer**; adapted and reproduced with permission from American Chemical Society [201]**. (A)** Synthesis of red blood cell membrane-coated nanoparticles (RBCMNPs) and their application in photothermal therapy and imaging. **(B)***In vivo* IR thermal images of tumor-bearing mice before and after treatment with different NPs. **(C)** MRI images of mice injected with different NPs. Tumor volume **(D)** and tumor weight **(E)** after treatment with different NPs.

Rao et al. [201] used co-extrusion (RBC-MNS-C) and microfluidic electroporation (RBC-MNS-E) to coat RBC films on magnetic Fe_3_O_4_-NPs. Owing to the excellent magnetic and photothermal properties of Fe_3_O_4_ and the long blood circulation characteristics of the RBCM, the bionic nanoDDS was used for tumor MRI and PTT (Fig. 11A). Fig. 11B shows the IR thermal images in mice, showing that the tumor temperature of mice treated with RBC-MNS ​+ ​laser can be significantly increased in 5 ​min (RBC-MNS-C: 34.6 ​°C–53.8 ​°C, RBC-MNS-E:34.5 ​°C–55.2 ​°C). The treatment effect was significantly better than that of PBS ​+ ​laser (34.4 ​°C–43.5 ​°C) and MNS ​+ ​laser groups (34.5 ​°C–49.3 ​°C). Fig. 11C shows T2-weighted MRI images of a tumor in mice before and after treatment. There was almost no change in mice injected with MNs, and for RBC-MNs-E, the tumor is obviously darkened. The results showed that the enrichment of RBC-MNS-E in tumors was better than that of RBC-MNA-C and MNS. Fig. 11D and E showed the change in tumor size. We can see that RBC-MNS-E has the best therapeutic effect, followed by RBC-MNS-C. All the results showed that NPs modified by cell membranes had better therapeutic effects, and microfluidic electroporation can protect the function of cell membrane better than co-extrusion.

Yang et al. [229] used the cell membrane (CM) of SGC7901 ​cells to modify the silicon dioxide NPs (SLN) loaded with the photodynamic reagent chlorine e6 (Ce6) (CM/SLN/Ce6). CM/SLN/Ce6 showed good dispersion and stability under physiological conditions, and could produce more ROS then SLN/Ce6 and free Ce6. *In vivo* results showed that CM/SLN/Ce6 was mainly involved in tumors, while SLN/Ce6 was mainly distributed in the liver and spleen. The most severe apoptosis was observed in the CM/SLN/Ce6 group, but no significant weight loss was observed in this group. These results demonstrated the tumor targeting ability of CM/SLN/Ce6, which had a better anticancer efficacy than LN/Ce6 and free Ce6, and reduced toxicity and side effects drugs due to good tumor homing and targeting.

### 6.3 Immunotherapy

Immunotherapy of cancer is a therapeutic method that uses drugs or biological agents to regulate the immune state of the body and elicit appropriate immune reaction, thus preventing and treating cancer. The TME is important in tumor immunity because it is closely related to the occurrence and development of tumor. The TME is usually characterized by vascular abnormalities, low pH, and hypoxia [230,231], and it is generally recognized that one of the biggest obstacles to antitumor immunotherapy is immunosuppression caused by the TME. Therefore, remodeling the TME can promote the infiltration of immune cells and inhibit tumor development and metastasis [232]. There are many methods of tumor immunotherapy, including monoclonal antibody therapy, immune checkpoint inhibitor therapy, adoptive cell therapy, oncolytic virus therapy, and tumor vaccines. However, the low accuracy of the delivery of drugs to tumors for immunotherapy is an important problem that need to be solved at present. The development of CMNPs has opened a new direction for tumor immunotherapy. The advantage of using CMNPs is that the patients' own tumor cells can be used to achieve precise personalized treatment, and the polymer core can also load multiple drugs to better adjust the antitumor immune response. In addition to coating immune drugs, cell membranes, such as those from WBC and NKC can secrete immune factors [184].

**Fig. 12:**
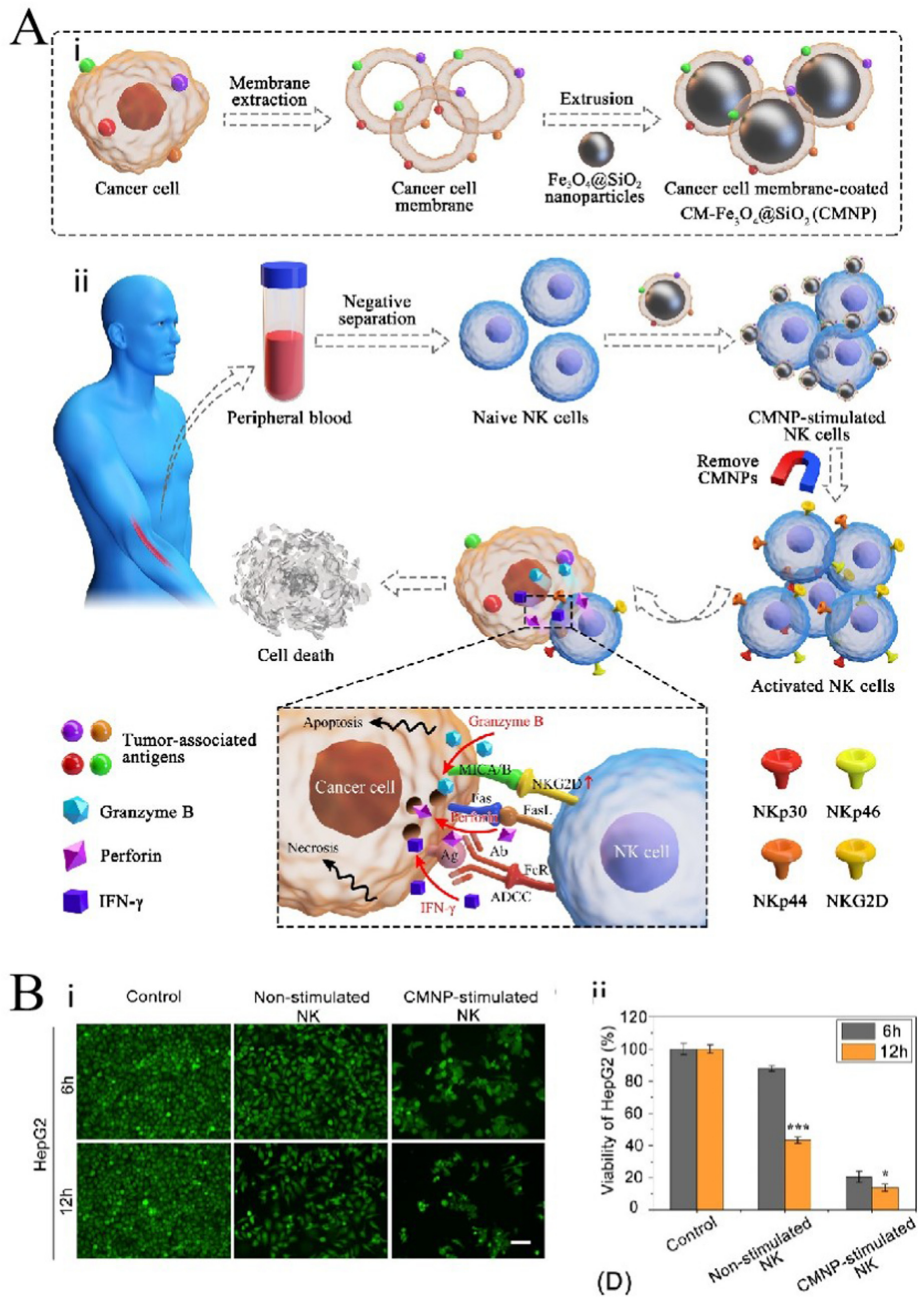
**Application of cell membrane-coated nanoparticles (CMNPs) in immunotherapy of cancer**; adapted and reproduced with permission from Elsevier [132]**. (A)** i. Composite diagram of CCMNPs. ii. CCMNPs activates NK cells to achieve tumor immunotherapy. **(B)** Calcein-AM staining results after treatment with different NPs.

Wu et al. [132] used CCMNPs to activate NK cells and achieve NK cell based tumor immunotherapy. Fig. 12A–i shows the synthesis process of the CCMNPs. The magnetic Fe_3_O_4_ NPs were modified using a silicon dioxide layer and then encapsulated in a cancer cell membrane. The CCMNPs can up-regulate the expression of NK cell surface-activated receptors and markers and induce the secretion of cytotoxic factors, thus enhancing the anti-tumor effect of NK cells (Fig. 12A–ii). The Calcein-AM staining showed that, compared to non-stimulated NK cells, the CNMP-stimulated NK cells showed a stronger tumor cell-killing effect (Figure B).

Tumor associated macrophages (TAMs) are important cells for tumor development, and M2-like TAMs can promote tumor immunosuppression and prolong the drug efficacy in the TME. Therefore, this is the main cell type that mainly promotes tumor development. On the contrary, M1-like TAMs have anti-tumor effects because they can secrete inflammatory cytokines [233,234]. The polarization of TAM to M1 or M2 depends on the signal molecules in the TME [235]. Chen et al. [236] wrapped the UPNPs with a TAM membrane and conjugated a photosensitizer to realized photodynamic immunotherapy for cancer with the synthetic CMNPs. This method changed the state of the TME and effectively transformed M2 macrophages into M1 macrophages and induced the production of tumor-specific effector T cells to enhance anti-tumor immune efficiency.

### 6.4 Gene therapy

In addition to the aforementioned methods for tumor treatment, CMNPs have also been used for tumor gene therapy. The gene therapy of cancer refers to the introduction of exogenous target genes into target cells through vectors to correct over-activated or defective genes, thus achieving the purpose of cancer therapy. However, the efficient and safe delivery of genes remains a key challenge for their clinical application [237]. Cell membranes can be used as carriers to deliver genes, because they can improve the circulation time of drugs and tumor targeting and can improve gene transfection efficiency. As early as 2010, Gao et al. [238] proposed and discussed the prospect of using MSCs as a targeted delivery vehicle for cancer gene therapy and constructed an SC-targeted vector carrying suicide genes between viral and non-viral gene transfection systems. In a mouse model of lung metastasis of melanoma, BMSCs (TK BMSCs) expressed by cytomegalovirus thymidine kinase (CMV TK) combined with the prodrug ganciclovir were delivered to the lung tissue through the bystander effect, thereby significantly inhibiting the growth of melanoma metastatic tumors and treating lung melanoma metastasis. The *in vitro* experimental results showed that engineered BMSCs had a significant suicide effect in the presence of ganciclovir [239].

**Fig. 13:**
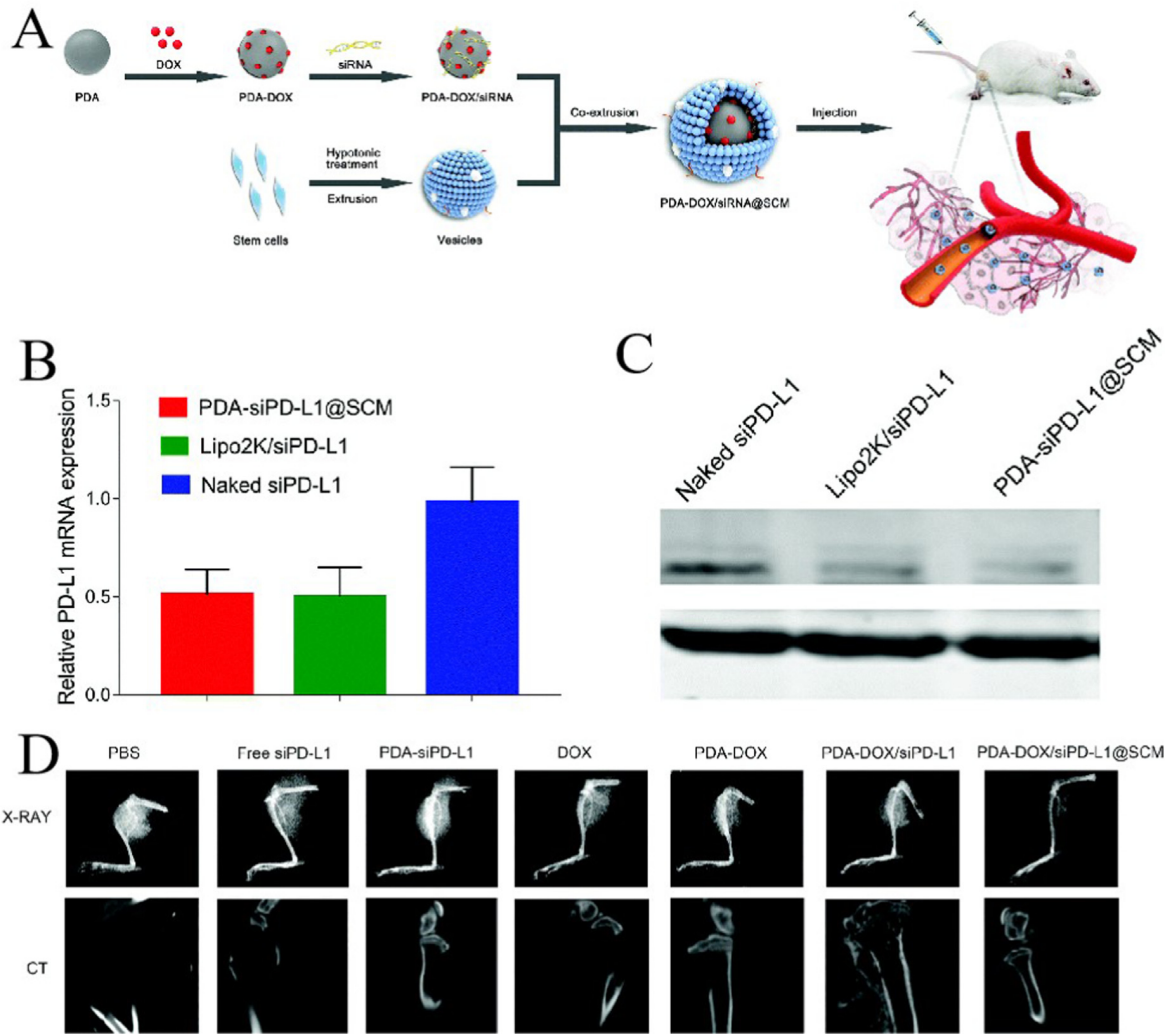
**Application of cell membrane-coated nanoparticles (CMNPs) in gene therapy of cancer**; adapted and reproduced with permission from Royal Society of Chemistry [180]**. (A)** Schematic diagram of synthesis of stem cell membrane-coated nanoparticles (SCMNPs) and treatment of bone metastasis of prostate cancer in mice. Detection of mRNA **(B)** and corresponding protein **(C)** expression by cell experiment. **(D)** Imaging images after different drug treatments.

Mu et al. [180] used a SC membrane (SCM) to wrap the NPs containing DOX and programmed cell death ligand 1 (PD-L1) siRNA (PDA-DOX/siPD-L1@SCM) to achieve the combine treatment of gene- and chemotherapy in a prostate cancer bone metastasis model (Fig. 13A). Fig. 13B (qRT-PCR) and Fig. 13C (western blot) show that the a better *in vitro* gene silencing effects of PDA-DOX/siPD-L1@SCM were better than those of naked siPD-1 and Lipo2k/siPD-1. Fig. 13D shows X-ray and computed tomography imaging after treatment. We can see for the X-ray imaging that there was almost no shadow indicating prostate cancer tissue in the PDA–DOX/siPD-L1@SCM treatment group, and the computed tomography showed that the bone cortex of the tibia was almost complete in the PDA–DOX/siPD-L1@SCM treatment group. However, evident tumor tissue shadow and bone cortex destruction can be seen in the other groups. All these results indicate that PDA – DOX/siPD-L1@SCM It can effectively inhibit the bone metastasis of prostate cancer and weaken the bone invasion of metastatic prostate cancer.

### 6.5 Other therapeutic methods

In addition to the above treatment methods, CMNPs are also used in other tumor treatments. Sonodynamic therapy uses ultrasound to penetrate biological tissues, gather sound energy in deep tissues, and activate sound sensitive drugs to produce anti-tumor effects. This method has a few side effects. Commonly used sonosensitizers include hematoporphyrin, photofrin, dihydroporphyrin and phthalocyanine. However, these sonosensitizers have low stability. But can be effectively protected by wrapping them with a cell membrane [240]. Research has shown that the iridium complex functionalized black-titanium NPs camouflaged by a cancer cell membrane can be used for PTT and sonodynamic therapy and tumor imaging, which the tumor was completely eradicated in a mouse model [97].

Chemodynamic therapy is a new type of tumor treatment technology based on the transformation of tumor endogenous chemical products (e.g., H_2_O_2_). This treatment was first promoted by Bu's group, which used Fenton or Fenton-like reactions to treat in situ cancer [241]. Researchers have prepared hollow manganese dioxide NPs coated with alendronate (ALD)/K7M2 cell membrane as nano-carriers to load ginsenoside Rh2 (Rh2), which is used for the MRI-guided immuno-chemodynamic therapy of osteosarcoma [242].

The role of CMNPs in tumor radiotherapy has been studied in recent years. Hypoxia is an important characteristic of the TME [230]. During radiotherapy, hypoxia may cause radiotherapy resistance, leading to tumor recurrence. Perfluorodecalin (FDC) has a high oxygen-carrying capacity and suitable half-life. Yu et al. [243] wrapped FDC with a RBCM, which protected FDC from emulsification and facilitated effective input in the tumor tissue. The results showed that compared with radiotherapy alone, this method can effectively reverse tumor hypoxia.

Magnetocaloric therapy kills tumor cells by converting magnetic energy into heat energy. It has the advantages of heat-inducing property, chemical stability, and targeting [244,245]. Cai et al. [246] used the cancer cell membrane to wrap the mesoporous silica NPs containing superparamagnetic ferroferric oxide and PTX, which effectively inhibited the growth of MDA-MB231 ​cells.

In the research on CMNPs for the diagnosis and treatment of tumors, tumor imaging is usually carried out together with other treatment methods. The targeting and imaging properties of CMNPs can more accurately and clearly display the location and shape of tumors [247]. Zhu et al. [248], they coated drug-loaded NPs (Fe3O4-DOX) with allogenic cancer cell membrane. The acquired MNP@DOX @CCCM is highly targeted to homologous tumors and wad effective for treating tumors and MR imaging.

## 7 Conclusion and outlook

This article focuses on CMNPs and their applications in tumor-targeted therapy. Membrane sources mainly include RBC, PLTs, WBC, cancer cells, and SCs. In addition, CMNPs have been applied to various materials such as polymers, gels, inorganic substances, and metals. Based on charge, hydrophobicity, structure, or size, the synthesis of nano-cores can be modified to accommodate almost any payload. Membranes from different source cells have different advantages and characteristics depending on the biological characteristics of the source cells, such as enzymes and ion channels. For example, RBCs have a long cycle time and cancer cells have tumor-targeting characteristics. Furthermore, cell membranes have immune evasion ability and functions such as crossing the blood-brain barrier and endothelium [249,250]. Moreover, the drug-loading capacity is relatively high, which can maximize the use of low-dose drugs, thus reducing organ toxicity. Two different biofilms can be combined through a fusion membrane to maximize the advantages. Through specific synthesis technologies, multiple functions can be organically integrated into the same NP. Cell membrane coating of NPs has innovatively enhanced the interaction between NPs and the biological environment, further developing tumor-targeted therapy methods from a biological perspective. The NPs support and stabilize the morphology of the cell membrane shell and simultaneously contain one or more types of small molecules or biomacromolecule therapeutic drugs. Such system have been studied in tumor-targeted chemotherapy, immunotherapy, PTT, and gene therapy and can be used to treat tumors from multiple levels through the preparation of cell membranes and NPs.

In addition to cell membranes, exosomes are also commonly used as biomimetic carriers of nanodrugs. Exosomes contain proteins, lipids and nucleic acids, giving them some advantages in cancer therapy. Han et al. [251] constructed biological NPs from the exosomes of M1 (M1-Exo) macrophages, used the inherent cytokines in M1-Exo to regulate the TME, and combined with PTT and gene therapy for tumor treatment. However, there are some disadvantages. It is difficult to deliver hydrophobic drugs and drugs with different physicochemical properties, and it is difficult to control drug release because of the small design space [184]. Moreover, tumor-derived- exosomes have potential tumor-promoting effects, because they contain some functional biological molecules, such as microRNA [252,253].

CMNPs have many clinical benefits in tumor treatment: 1) CMNPs have a better targeting ability and can deliver drugs to the tumor accurately, thus improving the therapeutic effect. 2) CMNPs have few side effects because they can deliver drugs to tumors more accurately. This can reduces the damage that drugs can cause to normal cells. 3) The cell membrane can protect the drugs from the influence of the external environment, thus improving drug stability. 4) CMNPs have higher biocompatibility and better targeting ability, which improve drug bioavailability, and can simulate the surface characteristics of normal cells to avoid being attacked by the immune system, thus reducing drug consumption and reducing treatment costs. 5) Some CMNPs can also be used for both imaging and prevention of specific tumors, realizing the integration of diagnosis, prevention and treatment of tumors. 6) NPs and cell membranes are diverse and are highly modifiable. Personalized CMNPs can be designed according diseases being targeted.

Despite of the benefits of CMNPs, there are also some limitations should be discussed, and they have not been applied in clinical practice. 1) The separation and purification of the cell membrane still needs to be adjusted and optimized. Although it is much easier to obtain cell membranes than exosomes, many cultured cells are still required to obtain cell membranes, and the preparation process is relatively complex and needs to be further simplified. For cancer cell membranes, the nucleus and genetic material should be strictly removed to eliminate potential carcinogenicity; RBCs and WBSs should be screened for blood group compatibility and infectious diseases [228]. Immortal cell-derived cell membranes may lead to unwanted biological effects. The expression of major histocompatibility complex molecules on the membrane of immune cells can cause immunogenicity problems. In addition, most studies have been conducted in mouse models, and whether CMNPs have potential side effects and long-term systemic toxicity in humans is unknown. 2) The prolonged circulation time and immune escape of CMNPs in *vivo* depend on the cell membrane and membrane proteins. Therefore, maintaining the integrity of cell membranes is important for CMNPs because cell membrane function is largely determined by cell integrity [29]. However, it is difficult to retain the intrinsic function of the membrane during the process of membrane extraction and storage. Complex, delicate, and sterile steps are involved, that may introduce multiple variability factors leading to changes in important characteristics of the NPs (such as purity and integrity), increasing the cost of CMNP preparation. Intrinsic proteins and ligands on the membrane are sensitive to exogenous and endogenous temperature, and inappropriate reaction and storage conditions may result in impaired integrity of membrane proteins. Moreover, long-term storage faces the potenticl for virus and pyrogen contamination, and the membrane protein may undergo immune reaction denaturation [[254], [255], [256]]. 3) Cells at different growth stages have different characteristics, and corresponding membrane and membrane proteins are also different, which may lead to batch-to-batch differences in the therapeutic effect of CMNPs [212,257]. 4) Membrane modifications used to enhance CMNPs may cause undesirable side effects. The overuse of CMNPs can cause or exacerbate inflammation through interaction with the immune system and lead to the release of pathological mediators. In addition, the modification process inevitably interferes with surface activity and blocks membrane proteins, and the function and activity of the modified cell membrane are difficult to evaluate [34]. If the structure or surface proteins of the cell membrane are destructed, then the immune system may recognize CMNPs as “foreign substances” and eliminate them. Hybrid membranes have also been a hot topic in recent years. It is important to ensure that the characteristics of the cell membranes are not damaged during preparation of the fused membrane. 5) A variety of proteins exist on the surface of CMNPs, but the function of all of them is not known at present. Identification of useful proteins and removal of irrelevant proteins may improve the therapeutic of CMNPs [258]. In addition, the mechanism of CMNPs in *vivo* has not been fully clarified, and whether the interaction between NPs and cell membrane can change the conformation of molecules has not been confirmed in most studies [257]. 6) The complex transport mechanism of different CMNPs *in vivo* is not fully understood. Research on CMNPs is still in the laboratory stage, and they have not been applied clinically. Thus and more experimental data are needed to prove their safety and effectiveness. A model system is crucial for discovering, developing, and testing new therapies for tumor diseases. Tumor-on-chip is a model system developed in recent years to study the progress of tumors [259]. Such models can be used to study the treatment of tumors by CMNPs. In addition, NPs modified by the cell membrane are ultimately targeted at the patients themselves, but most previous research focused on individual cells or non-human animal. Making the preparation safer for patients and reducing the drug price by reducing the production difficulty are also noteworthy goals. Although the use of CMNPs in the treatment of tumors has only begun to develop in recent years, it has developed rapidly, and many studies have been conducted on, for example, inflammation and cardiovascular disease, etc. [[260], [261], [262], [263]]. For the treatment of tumors, it has low harm to patients and fewer side effects. It can exert a greater antitumor effect when combined with other technologies, such as genetic engineering [264] and transgenic technology [162]. 7) There are many kinds of cancer and their effects are personalized to individual patients, and the clinical application of CMNPs currently lacks guidelines and medical-related laws.

In general, CMNPs have great advantages for tumor diagnosis and treatment, but most of them are still in the research stage. They will become a major trend in tumor treatment in the future.

## Credit authors statement

Qinglai Tang, Lanjie Lei and Shisheng Li designed research; Shiying Zeng, Minna Xiao, Xinying Tong, Tao Yang and Danhui Yin researched and wrote the paper. All the authors read and approved the final manuscript.

## Declaration of competing interest

The authors declare that they have no known competing financial interests or personal relationships that could have appeared to influence the work reported in this paper.

## Data Availability

No data was used for the research described in the article.
